# A landmark-based dynamic prediction model for anastomotic leakage after rectal cancer surgery: integrating perioperative and postoperative trajectories

**DOI:** 10.3389/fonc.2026.1915256

**Published:** 2026-09-16

**Authors:** Mingwei Luo, Jiemin Zhou, Yueshan Pang, Zhixiang Xing, Juncheng Yuan, Yifan Zhang, Jiebin Xie

**Affiliations:** 1 Department of Gastrointestinal Surgery, Affiliated Hospital of North Sichuan Medical College, Nanchong, Sichuan, China; 2 Department of Clinical Medicine, North Sichuan Medical College, Nanchong, Sichuan, China; 3 Department of General Practice, Affiliated Hospital of North Sichuan Medical College, Nanchong, Sichuan, China

**Keywords:** anastomotic leakage, dynamic risk stratification, inflammatory trajectory, landmark-based dynamic prediction model, machine learning, rectal cancer

## Abstract

**Background:**

Anastomotic leakage (AL) remains a severe complication after radical surgery for rectal cancer. Current prediction models rely mainly on static variables and may not capture evolving perioperative physiological stress. This study developed and validated a twostage landmark-based dynamic prediction model that integrates baseline clinical features with postoperative inflammatory trajectories for individualized AL risk stratification.

**Methods:**

We retrospectively analysed 815 patients who underwent radical rectal cancer resection. A consensus feature set was derived by majority voting across four feature-selection pipelines: LASSO, RF-MDA, SVM-RFE, and stepwise AIC. Model 1 used preoperative and intraoperative variables to estimate AL risk immediately after surgery. Model 2 sequentially incorporated early postoperative dynamic indicators. Model performance was assessed using the area under the receiver operating characteristic curve (AUC), calibration curves, integrated discrimination improvement (IDI), net reclassification improvement (NRI), and decision curve analysis (DCA).

**Results:**

AL occurred in 82 of 815 patients (10.1%). The multi-algorithm consensus strategy identified 16 core baseline variables. Model 1 showed strong discrimination, with an AUC of 0.825 (95% CI, 0.774–0.875). After incorporating early postoperative dynamic parameters, including longitudinal neutrophil-to-lymphocyte ratio trajectory clusters and acute hypoalbuminemia, Model 2 improved the AUC to 0.889 (95% CI, 0.848–0.930; DeLong test, *P <* 0.001). Model 2 also improved risk reclassification, with an IDI of 0.116 (*P <* 0.001) and a net NRI of 0.129 (*P* = 0.006). The Brier score decreased to 0.057, and DCA showed consistently greater clinical net benefit across a broad range of threshold probabilities.

**Conclusions:**

This two-stage landmark-based dynamic prediction framework improves early warning for AL by combining baseline risk factors with postoperative inflammatory dynamics. The model may support more precise perioperative risk stratification and earlier individualized intervention after radical rectal cancer surgery.

## 1 Introduction

Colorectal cancer remains a major public health burden, ranking third in incidence and second in cancer-related mortality worldwide (1). Rectal cancer contributes substantially to this burden, with a continuing increase in disease incidence in many regions. Radical resection, particularly total mesorectal excision (TME), remains central to curative treatment (2, 3). Advances in minimally invasive surgery and neoadjuvant therapy have expanded the use of sphincter-preserving surgery for low and middle rectal cancer (4, 5).

However, oncological resection with intestinal reconstruction still carries a clinically important risk of anastomotic leakage (AL). Despite improvements in surgical technique, stapling devices, and perioperative care, AL occurs in 2% to 30% of patients across published series. AL reflects a defect in anastomotic integrity that permits communication between intraluminal contents and extraluminal tissues. This process can lead to pelvic infection, pelvic sepsis, and systemic inflammatory response syndrome. AL is also associated with reoperation, longer hospitalization, and higher perioperative mortality (6, 7). In addition, the inflammatory environment associated with AL may worsen oncological outcomes by increasing local recurrence and reducing overall survival (8). In clinical practice, colorectal surgeons must often balance the benefits of sphincter preservation against the protective value of a diverting stoma. This decision has become more important as low rectal cancer increases and patients place greater value on postoperative quality of life (9). Accurate, individualized prediction of AL is therefore essential for tailoring surgical strategy and perioperative management. Existing prediction models, however, rely largely on static variables measured before or during surgery, including sex, anastomotic height, and ASA physical status (10–12). These models provide useful baseline estimates but cannot capture the evolving host response to surgical injury. Their ability to support individualized risk assessment may therefore be limited. Moreover, most existing studies have focused on baseline characteristics, leaving early postoperative physiological changes insufficiently explored (13, 14).

Emerging evidence suggests that longitudinal postoperative biomarker patterns, including inflammatory and metabolic trajectories, may provide incremental information beyond static clinical parameters (15–18). These dynamic signals reflect the real-time physiological response to surgical trauma and may identify early biological signs of anastomotic failure. A prediction framework that integrates information across preoperative, intraoperative, and early postoperative phases may therefore better match clinical decision-making. Such a framework can update risk estimates as new information becomes available. We hypothesized that adding early postoperative inflammatory trajectories and metabolic indicators to baseline risk factors would improve AL risk stratification. Specifically, we expected a two-stage sequential model to improve discrimination, calibration, and clinical utility compared with conventional static models.

In this study, we developed and validated a two-stage landmark-based dynamic prediction model using time-series clinical data from patients undergoing radical rectal cancer resection. The first stage integrated preoperative and intraoperative risk factors to estimate AL risk immediately after surgery. The second stage incorporated early postoperative physiological stress indicators to dynamically refine risk prediction. By aligning model construction with the sequential accumulation of perioperative information, this framework may support more precise risk stratification, individualized intervention, and shared decision-making.

## 2 Method

### 2.1 Study design and patient selection

This single-centre retrospective cohort study followed the Transparent Reporting of a Multivariable Prediction Model for Individual Prognosis or Diagnosis (TRIPOD) guidelines. To establish a rigorous temporal framework for surgical risk assessment, we adopted a two-stage landmark analysis design. Consecutive patients who underwent laparoscopic radical resection with sphincter-preserving rectosigmoidectomy (Dixon procedure) for rectal cancer at the Affiliated Hospital of North Sichuan Medical College between January 2019 and December 2024 were screened for eligibility (**Figure 1**).

**Figure 1:**
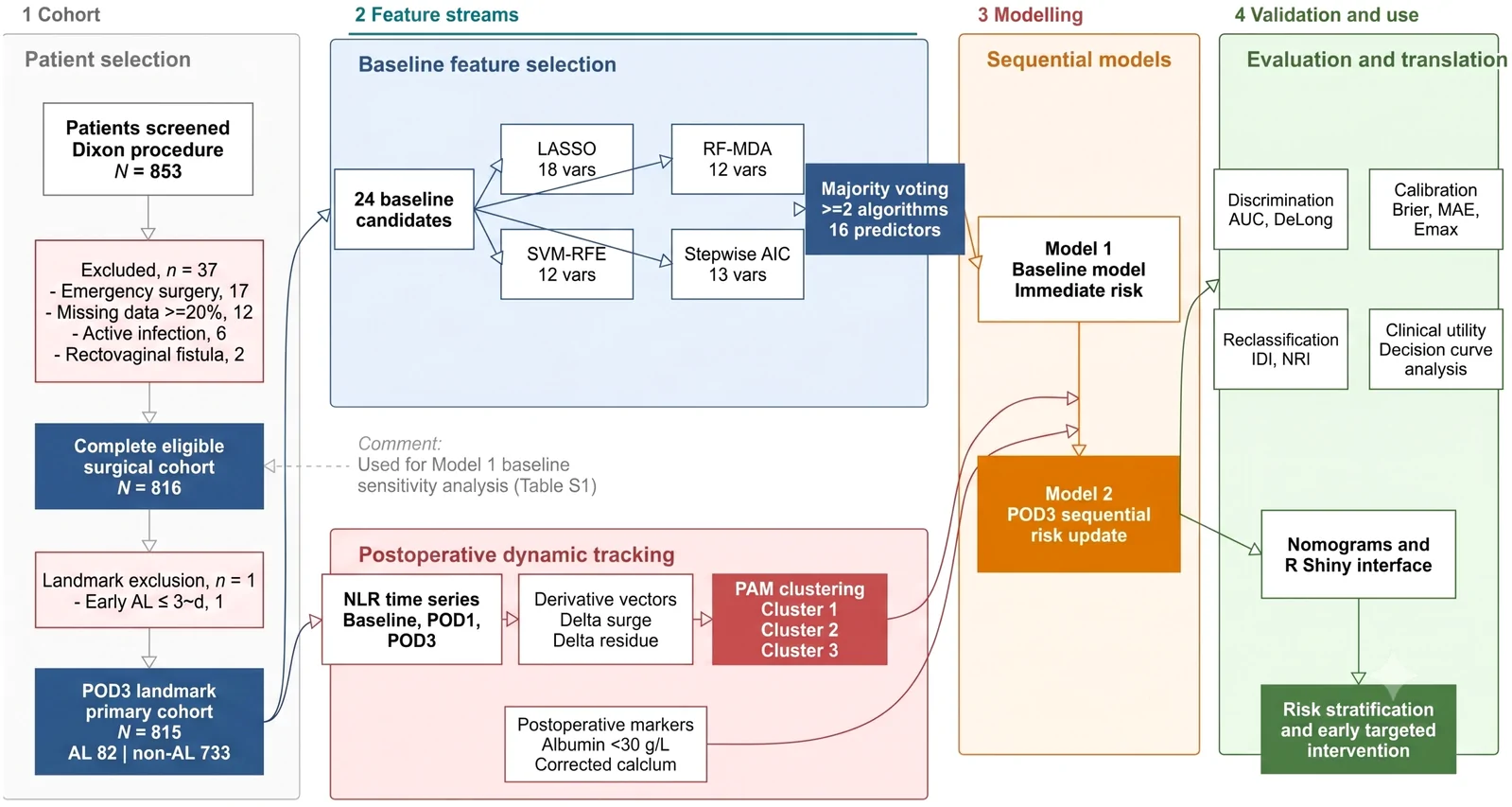
Flowchart of the two-stage landmark-based dynamic prediction model for anastomotic leakage (AL). (1) Cohort selection: From 853 screened patients,general exclusions yielded the complete eligible surgical cohort (*N* = 816). This complete cohort was specifically utilized for a sensitivity analysis to verify the baseline stability of Model 1 immediately after surgery (**Supplementary Table 1**). Subsequently, landmark exclusion of early AL diagnosed ≤ POD3 formally established the POD3 landmark primary cohort (*N* = 815). (2) Feature streams: Sixteen consensus baseline predictors were identified from 24 candidates using a majority voting strategy across four feature selection algorithms (LASSO, RF-MDA, SVM-RFE, and Stepwise AIC). Concurrently, postoperative longitudinal NLR trajectories were stratified via PAM clustering, alongside the tracking of dynamic markers (albumin <30 g/L, corrected calcium). (3) Modelling: Model 1 estimated immediate postoperative AL risk utilizing baseline predictors. Model 2 dynamically updated this risk on postoperative day 3 (POD3) by integrating early postoperative tracking variables. (4) Validation and translation: Model performance was rigorously assessed via discrimination, calibration, reclassification, and clinical utility metrics, culminating in the deployment of nomograms and an R Shiny web interface for individualized risk stratification.

Patients were eligible if they were adults (≥18 years) with histologically confirmed rectal adenocarcinoma undergoing elective curative-intent rectal resection with primary colorectal anastomosis. Exclusion criteria included emergency surgery, multiple primary colorectal cancers, concomitant rectovaginal or rectourethral fistulas, incomplete clinical data, active systemic infection, and severe comorbid conditions or physiological abnormalities that could substantially affect perioperative outcomes. Detailed exclusion definitions are provided in the **Supplementary Methods**. After applying the eligibility criteria, 816 patients were included in the final cohort.

We then excluded patients diagnosed with early anastomotic leakage within 3 postoperative days (≤ POD3) to construct the POD3 landmark primary cohort (*N* = 815). Because the core study objective required paired statistical tests for incremental predictive value (IDI, NRI, and the DeLong test), which depend on identical cohorts across the compared models, this landmark cohort served as the unified dataset for all model development (Model 1 and Model 2), internal validation, and performance comparisons in the main manuscript. The outcome prediction window was defined as commencing after POD3.

To confirm that the landmark exclusion step did not introduce selection bias into the baseline predictor estimates, we performed a sensitivity analysis of Model 1 on the complete eligible surgical cohort (*N* = 816). Predictor distributions and coefficients remained consistent between the two cohorts (**Supplementary Table 1**), confirming that the baseline risk estimates derived from the landmark cohort are robust and generalisable to the full surgical population.

To minimise inter-surgeon variability, all procedures were performed by experienced colorectal surgeons following strictly standardised institutional operative protocols. The primary outcome was AL within 30 days after surgery. AL was diagnosed and graded according to the International Study Group of Rectal Cancer (ISREC) consensus criteria. Clinical variables were independently extracted and cross-checked by two researchers.Missing values for candidate predictors were addressed using multivariate imputation by chained equations (MICE) to generate a single fully imputed dataset for all downstream modeling.

### 2.2 Longitudinal NLR trajectory clustering

The neutrophil-to-lymphocyte ratio (NLR) was used as the core longitudinal biomarker of early postoperative systemic inflammation. NLR values were collected at preoperative baseline, postoperative day 1 (POD1), and postoperative day 3 (POD3). Two dynamic derivative vectors were then generated:

-
${\text{ΔNLR}}_{\text{Surge}}={\text{NLR}}_{\text{POD}1}-{\text{NLR}}_{\text{Baseline}}$, representing the acute inflammatory response to surgical trauma;
-
${\text{ΔNLR}}_{\text{Residue}}={\text{NLR}}_{\text{POD}3}-{\text{NLR}}_{\text{Baseline}}$, representing persistent inflammatory burden or delayed inflammatory resolution during early recovery.

After Z-score standardisation, these two vectors were entered into an unsupervised Partitioning Around Medoids (PAM;K-medoids) clustering algorithm. Anastomotic leakage outcomes were masked during cluster derivation to prevent information leakage.The optimal number of clusters was selected using the within-cluster sum of squares (WSS) elbow method, the average silhouette coefficient, and clinical interpretability. The silhouette coefficient favoured *K* = 2, while the WSS curve showed a sustained deceleration at *K* = 3. A three-cluster solution was further motivated by the expectation of three clinically distinct inflammatory phenotypes: Cluster 1 (stable/resolution), Cluster 2 (acute hyper-response) and Cluster 3 (delayed prolonged-risk) Because a two-cluster solution would obscure the highest-risk phenotype, *K* = 3 was selected for the final clustering solution (**Supplementary Figures 1**-**3**).

Cluster structural stability was confirmed via bootstrap resampling. The fitted scaling parameters, robust medoid coordinates, and minimum Euclidean distance assignment rules for prospectively classifying new patients are provided in **Supplementary Table 2**. Sensitivity analyses using *K* = 2 and continuous longitudinal NLR modelling confirmed that *K* = 3 offered the best clinical risk stratification.

Postoperative serum calcium concentrations were corrected for perioperative hypoalbuminaemia using the Payne formula (Equation 1):

(1) Corrected calcium (mmol/L)=measured total calcium (mmol/L)+0.02×[40 −serum albumin (g/L)]

For patients with serum albumin ≥ 40 g/L, the measured total serum calcium concentration was used without correction.

### 2.3 Ensemble feature selection and two-stage sequential model construction

A candidate set of 24 preoperative and intraoperative variables, covering demographics, comorbidities, baseline laboratory results, oncological characteristics, and surgical parameters, was analysed. To mitigate multicollinearity and enhance feature stability, the variable importance and ranking profiles of these predictors were evaluated using a multi-algorithm ensemble voting strategy (**Supplementary Figures 10**-**13**) based on four distinct frameworks:

- **LASSO regression (glmnet):** $L_{1}$-regularised binomial logistic regression with the optimal penalty parameter ( $\lambda_{\text{min }}$) selected by 10-fold cross-validation minimising binomial deviance over a 100-point grid. Variables with non-zero coefficients were retained.
- **Random forest (randomForest):** 500 trees; variable importance assessed by mean decrease accuracy (MDA). Predictors ranking in the top 50% were retained.
- **SVM-RFE (caret, kernlab):** Linear-kernel support vector machine with recursive feature elimination guided by squared feature weights, embedded in 5-fold cross-validation. Features were eliminated until maximum classification accuracy was reached.
- **Stepwise regression (MASS):** Bidirectional selection minimising the Akaike information criterion (AIC).

The outputs from these four methods were combined using a majority voting strategy. Variables selected by at least two algorithms were retained as consensus predictors.

A two-stage landmark-based dynamic prediction system was then constructed to reflect the chronological accumulation of perioperative information. Model 1 was fitted using multivariable logistic regression with the consensus preoperative and intraoperative predictors. This model provided an immediate AL risk estimate immediately after surgery.

Model 2 extended Model 1 by incorporating early postoperative dynamic variables: NLR trajectory subtype, postoperative severe hypoalbuminaemia (albumin *<* 30 g/L), and corrected serum calcium on POD3. Cluster 1 was used as the reference category for the NLR trajectory variable. Both models were visualised as static nomograms and deployed as web-based interactive prediction interfaces.

### 2.4 Model evaluation and validation

Model discrimination was assessed using the area under the receiver operating characteristic curve (AUC). Differences in AUC between the two sequential models were compared with the DeLong test.

Optimism-corrected internal validation was performed using 1,000 bootstrap resamples to evaluate model stability. Because repeating the full multi-algorithm feature selection pipeline within each resample was computationally impractical, the bootstrap procedure focused on assessing the stability of coefficient estimation and risk calibration for the final predictor set. This set was derived from the majority voting strategy. The structural stability of the predictor set itself was verified through a separate bootstrap feature selection frequency analysis over 200 iterations,yielding a calibration slope of 0.7963. Potential overfitting given the number of events was examined by evaluating the effective parameter complexity (22 degrees of freedom) against the Riley sample size criteria for prediction models. An optimism-corrected calibration slope was derived and applied as a uniform shrinkage factor to penalise the final regression coefficients.

Calibration was assessed using bootstrap bias-corrected calibration curves. Prediction error was quantified with the Brier score, mean absolute error (MAE), and maximum absolute error (*E*_max_).

The incremental value of postoperative dynamic variables was evaluated using net reclassification improvement (NRI) and integrated discrimination improvement (IDI), with 95% confidence intervals. NRI was calculated at a prespecified clinical risk threshold of 10%. Clinical net benefit was assessed across threshold probabilities using decision curve analysis (DCA). To maintain consistency across baseline tables, multivariable forest plots, and calibration plots, All regression analyses and model-fitting workflows were performed using the fully imputed analytical dataset generated by multivariate imputation by chained equations (MICE) (N = 815). All statistical analyses were performed in R. A two-sided *P <* 0.05 was considered statistically significant.

## 3 Results

### 3.1 Patient demographics and univariate analysis

A total of 815 patients who underwent laparoscopic radical resection with sphincter-preserving rectosigmoidectomy (Dixon procedure) for rectal cancer were included (**Table 1**). Within 30 days after surgery, anastomotic leakage (AL) occurred in 82 patients, corresponding to an incidence of 10.1% (82*/*815). The median age was 66.0 years (interquartile range [IQR], 55.5–73.0 years). The cohort included 534 men (65.5%) and 281 women (34.5%).

**Table 1:** Baseline perioperative demographics, clinical-pathological characteristics, and longitudinal parameters of the patient cohort stratified by the occurrence of anastomotic leakage.

Characteristic	Total (n=815)	Non-AL (n=733)	AL (n=82)	*P*-value
Preoperative characteristics				
Sex (%)				0.001
Female	281 (34.5)	266 (36.3)	15 (18.3)	
Male	534 (65.5)	467 (63.7)	67 (81.7)	
Age, years	66.00 [55.50, 73.00]	66.00 [55.00, 73.00]	64.00 [57.00, 73.00]	0.759
BMI, kg/m^2^	23.03 [20.91, 25.04]	22.96 [20.83, 25.01]	23.42 [21.18, 25.08]	0.356
Hypertension (%)				0.009
No	624 (76.6)	571 (77.9)	53 (64.6)	
Yes	191 (23.4)	162 (22.1)	29 (35.4)	
Diabetes mellitus (%)				0.017
No	761 (93.4)	690 (94.1)	71 (86.6)	
Yes	54 (6.6)	43 (5.9)	11 (13.4)	
CAD (%)				0.772
No	780 (95.7)	702 (95.8)	78 (95.1)	
Yes	35 (4.3)	31 (4.2)	4 (4.9)	
Smoking history (%)				1
No	593 (72.8)	533 (72.7)	60 (73.2)	
Yes	222 (27.2)	200 (27.3)	22 (26.8)	
Alcohol consumption (%)				0.049
No	636 (78.0)	565 (77.1)	71 (86.6)	
Yes	179 (22.0)	168 (22.9)	11 (13.4)	
Prior abdominal surgery (%)				0.003
No	646 (79.3)	592 (80.8)	54 (65.9)	
Yes	169 (20.7)	141 (19.2)	28 (34.1)	
NAC (%)				0.069
No	668 (82.0)	607 (82.8)	61 (74.4)	
Yes	147 (18.0)	126 (17.2)	21 (25.6)	
Clinical stages (%)				0.004
1	29 (3.6)	28 (3.8)	1 (1.2)	
2	166 (20.4)	160 (21.8)	6 (7.3)	
3	539 (66.1)	473 (64.5)	66 (80.5)	
4	81 (9.9)	72 (9.8)	9 (11.0)	
ASA grade (%)				0.001
I–II	530 (65.0)	491 (67.0)	39 (47.6)	
III–IV	285 (35.0)	242 (33.0)	43 (52.4)	
Preop ALB (%), g/L				0.61
≥30g/L	805 (98.8)	723 (98.6)	82 (100.0)	
<30g/L	10 (1.2)	10 (1.4)	0 (0.0)	
Preop Hb, g/L	124.00 [111.00, 136.00]	124.00 [112.00, 136.00]	117.50 [108.00, 132.00]	0.013
CEA, ng/mL	2.34 [1.37, 4.94]	2.26 [1.35, 4.72]	3.44 [1.64, 7.04]	0.012
CA19-9, U/mL	12.40 [7.06, 22.38]	12.97 [7.20, 22.54]	9.35 [5.93, 15.43]	0.011
Preop corrected Ca, mmol/L	2.24 [2.17, 2.31]	2.25 [2.18, 2.31]	2.20 [2.09, 2.25]	<0.001
Tumor size, cm	3.70 [2.80, 4.80]	3.60 [2.70, 4.70]	4.00 [3.05, 5.00]	0.015
Distance from AV, cm	6.00 [4.00, 11.00]	7.00 [4.00, 12.00]	6.00 [5.00, 7.88]	0.017
Intraoperative characteristics				
Operative time, min	175.20 [145.20, 220.20]	175.20 [145.20, 214.80]	190.20 [166.20, 235.65]	0.003
EBL, mL	50.00 [40.00, 100.00]	50.00 [30.00, 100.00]	86.00 [50.00, 100.00]	0.011
Diverting stoma (%)				0.001
No	576 (70.7)	505 (68.9)	71 (86.6)	
Yes	239 (29.3)	228 (31.1)	11 (13.4)	
Blood transfusion				0.532
No	785 (96.3)	707 (96.5)	78 (95.1)	
Yes	30 (3.7)	26 (3.5)	4 (4.9)	
LCA preservation (%)				0.193
No	724 (88.8)	655 (89.4)	69 (84.1)	
Yes	91 (11.2)	78 (10.6)	13 (15.9)	
Postoperative characteristics				
NLR trajectory cluster (%)				<0.001
1	433 (53.1)	401 (54.7)	32 (39.0)	
2	260 (31.9)	244 (33.3)	16 (19.5)	
3	122 (15.0)	88 (12.0)	34 (41.5)	
Postop ALB (%), g/L				<0.001
≥30g/L	736 (90.3)	677 (92.4)	59 (72.0)	
<30g/L	79 (9.7)	56 (7.6)	23 (28.0)	
Postop corrected Ca, mmol/L	2.25 [2.17, 2.33]	2.25 [2.17, 2.33]	2.24 [2.16, 2.32]	0.319

ALB, albumin; AL, anastomotic leakage; ASA, American Society of Anesthesiologists; AV, anal verge; BMI, body mass index; Ca, calcium; CAD, coronary artery disease; CA19-9, carbohydrate antigen 19-9; CEA, carcinoembryonic antigen; EBL, estimated blood loss; Hb, hemoglobin; IQR, interquartile range; LCA, left colic artery; NAC, neoadjuvant chemotherapy; NLR, neutrophil-to-lymphocyte ratio. Categorical variables are presented as absolute numbers accompanied by percentages in parentheses: n (%). All continuous variables are non-normally distributed as evaluated by the Kolmogorov-Smirnov test and are uniformly expressed as Median [Interquartile Range] unless specified otherwise. Baseline significance testing was executed via the Mann-Whitney U test for continuous vectors and the Pearson Chi-square test or Fisher’s exact test for categorical proportions. (*P <* 0.05 indicates statistical significance).

As shown in **Table 1**, several perioperative characteristics differed between the AL and non-AL groups. Patients with AL were more often male (81.7% vs. 63.7%; *P* = 0.001) and more often lacked a diverting stoma (86.6% vs. 68.9%; *P* = 0.001). They also had higher rates of hypertension (35.4% vs. 22.1%; *P* = 0.009), diabetes mellitus (13.4% vs. 5.9%; *P* = 0.017), previous abdominal surgery (34.1% vs. 19.2%; *P* = 0.003), ASA grade III–IV (52.4% vs. 33.0%; *P* = 0.001), and advanced clinical tumour stage (*P* = 0.004). Alcohol consumption was less frequent in the AL group (13.4% vs. 22.9%; *P* = 0.049). For continuous and laboratory variables, patients with AL had a shorter distance from the tumour to the anal verge (median, 6.0 cm vs. 7.0 cm; *P* = 0.017), longer operative time (median, 190.2 min vs. 175.2 min; *P* = 0.003), and greater estimated blood loss (median, 86.0 mL vs. 50.0 mL; *P* = 0.011). The AL group also had lower preoperative corrected serum calcium (median, 2.20 mmol/L vs. 2.25 mmol/L; *P <* 0.001) and haemoglobin levels (median, 117.50 g/L vs. 124.00 g/L; *P* = 0.013). Maximum tumour size (median, 4.00 cm vs. 3.60 cm; *P* = 0.015) and baseline carcinoembryonic antigen concentrations (median, 3.44 ng/mL vs. 2.26 ng/mL; *P* = 0.012) were higher in the AL group. Baseline CA19–9 also differed between groups (median, 9.35 U/mL vs. 12.97 U/mL; *P* = 0.011).

### 3.2 Longitudinal postoperative inflammatory trajectory clustering

To characterise early postoperative inflammatory patterns, unsupervised PAM clustering was applied to the dynamic NLR derivative vectors, ΔNLR_Surge_ and ΔNLR_Residue_. This analysis identified three postoperative inflammatory trajectories (**Supplementary Figure 1**).

Cluster 1, designated as the stable resolution phenotype, comprised 433 patients (53.1% overall; 401 patients [54.7%] in the non-AL group and 32 patients [39.0%] in the AL group). This trajectory exhibited limited early post-traumatic stress responsiveness paired with rapid, natural clearance of inflammatory mediators by postoperative day 3 (POD3). Cluster 1 was established as the clinical reference baseline in subsequent multivariable regression workflows. Cluster 2, the stress responsive phenotype, included 260 patients (31.9% overall; 244 patients [33.3%] in the non-AL group and 16 patients [19.5%] in the AL group), demonstrating a moderate immediate post-surgical inflammatory surge followed by a swift decline by POD3. Cluster 3, the delayed prolonged-risk phenotype, encapsulated 122 patients (15.0% overall; 88 patients [12.0%] in the non-AL group and 34 patients [41.5%] in the AL group), and was characterized by a violent acute stress burst on POD1 coupled with substantial resolution failure and high residual inflammatory burden at POD3.

Univariate interaction mapping verified that the distribution of these three trajectory phenotypes was profoundly coupled with the occurrence of postoperative AL (*P <* 0.001).

### 3.3 Development of the two-stage landmark-based dynamic prediction system

A two-stage landmark-based dynamic prediction system was developed to evaluate perioperative predictors of anastomotic leakage (AL) (**Supplementary Tables 5**, **6**). Model 1 was constructed at surgical completion using 16 baseline variables (**Table 2**) In this baseline model, male sex, higher ASA grade, previous abdominal surgery, shorter distance from the tumour to the anal verge, longer operative time, and lower preoperative haemoglobin were independently associated with higher odds of AL. By contrast, diverting stoma formation, higher preoperative corrected calcium, older age, and alcohol consumption were associated with lower odds of AL.In the 200-iteration bootstrap selection frequency analysis, nine predictors achieved 100% selection (diverting stoma, ASA classification, preoperative corrected calcium, sex, prior abdominal surgery, alcohol consumption, tumour distance, NLR Cluster 3, and postoperative hypoalbuminaemia); age was selected in 99.5% of resamples.

**Table 2:** Preoperative and intraoperative variables selected by the four independent statistical and machine learning algorithms.

Method	Selected variables
LASSO	Sex, Diverting stoma, ASA, Preoperative corrected calcium, Distance from anal verge, Operative time, Preoperative hemoglobin, Neoadjuvant chemotherapy, Prior abdominal surgery, Alcohol consumption, Clinical stage, Hypertension, Age, Tumor size, CEA, LCA preservation, Diabetes mellitus, Coronary artery disease (CAD)
Stepwise AIC	Sex, Diverting stoma, ASA, Preoperative corrected calcium, Distance from anal verge, Operative time, Preoperative hemoglobin, Neoadjuvant chemotherapy, Prior abdominal surgery, Alcohol consumption, Clinical stage, Hypertension, Age
RF-MDA	Sex, Diverting stoma, ASA, Preoperative corrected calcium, Distance from anal verge, Operative time, Preoperative hemoglobin, Neoadjuvant chemotherapy, Prior abdominal surgery, Alcohol consumption, Estimated blood loss (EBL), Intraoperative blood transfusion
SVM-RFE	Sex, Diverting stoma, ASA, Preoperative corrected calcium, Distance from anal verge, Operative time, Preoperative hemoglobin, Clinical stage, Tumor size, Estimated blood loss, CEA, CA19-9

AIC, Akaike Information Criterion; ASA, American Society of Anesthesiologists; AV, anal verge; Ca, calcium; CAD, coronary artery disease; CEA, carcinoembryonic antigen; EBL, estimated blood loss; Hb, hemoglobin; LASSO, Least Absolute Shrinkage and Selection Operator; L1, regularized; MDA, Mean Decrease Accuracy; NAC, neoadjuvant chemotherapy; RF, Random Forest; SVM-RFE, Support Vector Machine-Recursive Feature Elimination. This selection matrix details the specific subsets of the 24 candidate preoperative and intraoperative features retained by each individual algorithmic framework. Features concurrently captured by two or more independent methodologies were sub-selected as the consensus core predictors through the predefined majority voting strategy to construct the baseline model (Model 1).

Model 2 further incorporated three early postoperative dynamic indicators. After adjustment for baseline variables, NLR trajectory Cluster 3, representing the delayed prolonged-risk phenotype, showed the strongest association with AL (aOR, 9.93; 95% CI, 4.69–21.04; *P <* 0.001). Severe postoperative hypoalbuminaemia was also independently associated with AL (aOR, 5.22; 95% CI, 2.36–11.54; *P <* 0.001). After dynamic indicators were added, the effects of operative time and preoperative haemoglobin were attenuated, whereas neoadjuvant chemotherapy became independently associated with AL (aOR, 2.49; 95% CI, 1.17–5.28; *P* = 0.018). Several baseline predictors remained stable, including male sex and ASA grade, while the association between diverting stoma formation and lower AL risk became stronger. The model structures are shown in **Figure 2**.

**Figure 2:**
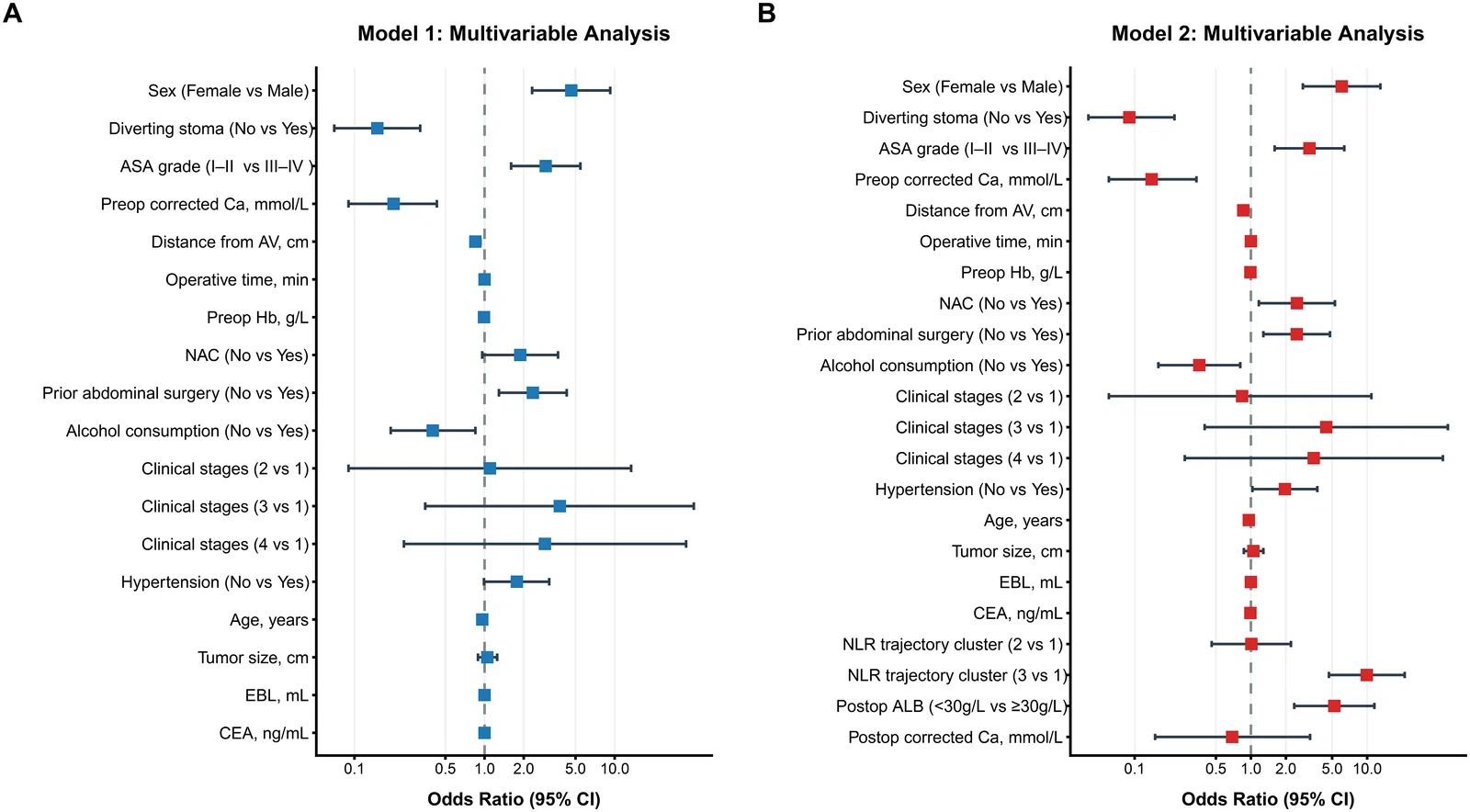
Forest plots of multivariable logistic regression analyses for the prediction models. **(A)** Model 1 (baseline risk assessment): Adjusted odds ratios (aORs) for the 16 consensus preoperative and intraoperative predictors. **(B)** Model 2 (sequential risk refinement): Updated risk profile on postoperative day 3 (POD3) integrating longitudinal trajectory and dynamic biological markers. Centers and error bars represent aORs with 95% confidence intervals (CIs). The vertical dashed line indicates an OR of 1.0. P *<* 0.05 indicates statistical significance.

### 3.4 Nomogram and web-based interface deployment

To maximize predictive accuracy, the final sequential logistic regression models retained the complete set of 16 consensus baseline predictors for Model 1, and 19 predictors for Model 2. Given the structural complexity of these comprehensive models, they were deployed as a user-friendly, interactive R Shiny web interface: https://nsmc-al-prediction-model.shinyapps.io/AL_Predictor_Web/. This digital platform serves as the primary clinical tool, outputting both raw probabilities and rigorously penalized risk estimates (adjusted via an optimism-corrected calibration slope of 0.7963) to support transparent and safe clinical decision-making.

Concurrently, to provide a visually interpretable alternative for rapid bedside assessment when the digital calculator is inaccessible, we constructed parsimonious visual nomograms. To prevent visual clutter and enhance clinical practicability, we purposefully performed a secondary feature reduction strictly for these static graphics. Specifically, the Model 1 nomogram illustrates only the top 8 most influential predictors (e.g., diverting stoma, ASA grade, and preoperative corrected calcium) selected from the full baseline set based on their coefficient magnitudes (**Supplementary Figures 14**-**16**).

Similarly, for dynamic risk updating on postoperative day 3 (POD3), the visual representation of Model 2 was condensed to 9 core parameters. This simplified Model 2 nomogram clearly demonstrates that NLR trajectory Cluster 3 and postoperative hypoalbuminaemia contribute the most significant weightings to the updated risk estimation (**Supplementary Figures 14**-**16**). We emphasize that while these condensed nomograms offer a convenient visual triage alternative, the comprehensive web-based calculator remains the definitive, internally validated tool for exact individualised risk calculation.

### 3.5 Model performance and incremental value

Head-to-head comparison showed that adding postoperative dynamic variables improved predictive performance (**Supplementary Tables 1**, **2**). Model 1 achieved an AUC of 0.825 (95% CI, 0.774–0.875). Model 2 increased the AUC to 0.889 (95% CI, 0.848–0.930). The DeLong test showed a significant improvement in discrimination (ΔAUC = 0.064; *P <* 0.001). Bootstrap analysis showed improved reclassification after adding longitudinal postoperative variables. Model 2 achieved an integrated discrimination improvement of 0.1157 (95% CI, 0.0760–0.1616; *P <* 0.001). At the prespecified 10% clinical intervention threshold, the net reclassification improvement was 0.1289 (95% CI, 0.0404–0.2179; *P* = 0.006). Decision curve analysis showed that Model 2 provided greater net benefit than Model 1 and default intervention strategies across threshold probabilities of 5% to 60% (**Figure 3**). After 1,000 bootstrap resamples, Model 1 had a mean absolute error of 2.28% and a maximum absolute error of 17.64%, mainly in the high-risk range above 60%. Model 2 showed lower prediction error, with a Brier score of 0.0570, mean absolute error of 1.14%, and maximum absolute error of 6.82%. Its calibration curve showed closer agreement with the ideal reference line (**Supplementary Figures 4**–**9**).

**Figure 3:**
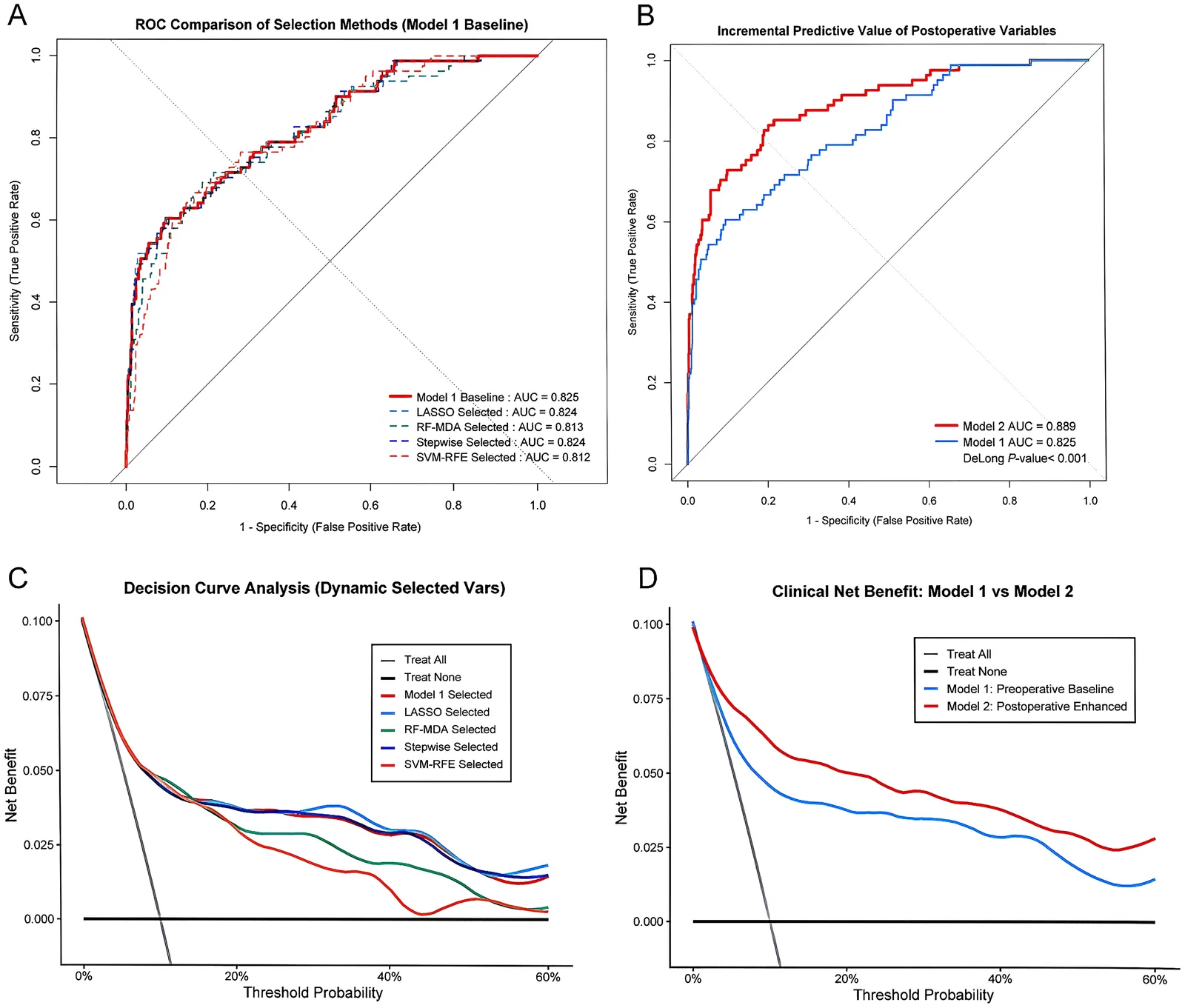
Discriminative performance and clinical utility of the machine learning algorithms and the two-stage prediction models. **(A)** Receiver operating characteristic (ROC) curves comparing the discriminative ability of the four individual machine learning algorithms and the consensus baseline model (Model 1). **(B)** ROC curves demonstrating the superior discrimination of the enhanced postoperative model (Model 2) compared to Model 1 (DeLong test, *P <* 0.001). **(C)** Decision curve analysis (DCA) evaluating the clinical net benefit of the initial algorithms and Model 1. **(D)** DCA illustrating the increased clinical net benefit of Model 2 over Model 1 across a wide range of threshold probabilities.

## 4 Discussion

In this study, we developed and validated a perioperative two-stage sequential model for dynamic prediction of anastomotic leakage (AL) after radical rectal cancer resection (19). The baseline model, which included preoperative and intraoperative variables, showed good discrimination (AUC: 0.825; 95% CI, 0.774–0.875). After adding early postoperative inflammatory trajectories and metabolic indicators, Model 2 achieved higher discrimination (AUC: 0.889; 95% CI, 0.848–0.930; DeLong test, *P <* 0.001). Calibration also improved, with *E*_max_ decreasing from 17.64% to 6.82% and the Brier score decreasing from 0.0657*to*0.0570. These findings suggest that AL is not determined only by baseline characteristics (20). Rather, it may evolve with the host’s perioperative physiological response (21). A sequential model may therefore support earlier risk updating before overt clinical deterioration occurs (22).

Most existing AL prediction models rely on a single modelling strategy, such as stepwise regression or LASSO (23). Such approaches may be sensitive to data noise, model instability, and local feature-selection patterns (24). To improve feature stability, we used an ensemble voting strategy that combined LASSO, RFMDA, SVM-RFE, and stepwise AIC (25). Seven predictors were retained by all four methods: sex, diverting stoma, ASA physical status, preoperative corrected calcium, distance from the anal verge, operative time, and preoperative haemoglobin (**Figure 4**). These variables represent several clinically plausible domains, including anastomotic anatomy, physiological reserve, surgical complexity, and tissue healing capacity (26, 27). Their consistent selection across different algorithms supports their role as stable baseline predictors in this cohort.

**Figure 4:**
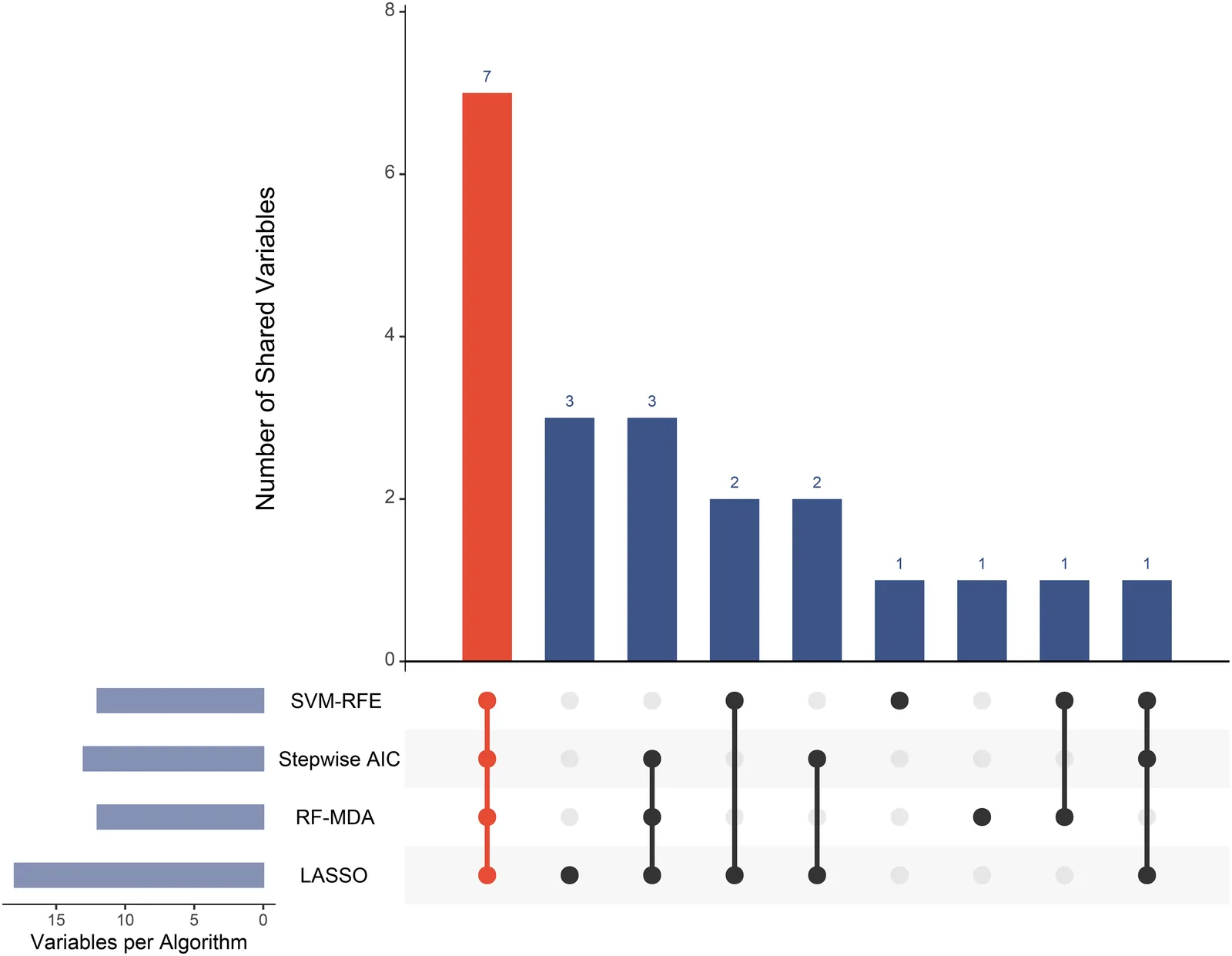
Consensus of variable selection across four distinct algorithms visualized by an UpSet plot. This UpSet plot displays the intersection sizes (top bar chart) and set sizes (left horizontal bar chart) of variables selected by four different feature selection techniques: Stepwise AIC, Support Vector Machine-Recursive Feature Elimination (SVM-RFE), Random Forest-Mean Decrease Accuracy (RFMDA), and Least Absolute Shrinkage and Selection Operator (LASSO). (Top Bar Chart) Shows the number of variables unique to specific combinations of selection methods. The leftmost bar confirms that 7 key variables (including Cluster NLR, Age, etc.) were consistently selected by all four algorithms. (Left Horizontal Bar Chart) Shows the total number of variables selected by each individual algorithm. (Matrix) The dark circles and lines connect the specific combinations of algorithms forming each intersection.

A key finding was the independent warning value of early postoperative inflammatory resolution. Conventional approaches often use single postoperative measurements of C-reactive protein or NLR (28). However, single time-point values may be affected by baseline immune status and variation in surgical trauma (29). By modelling NLR changes from baseline to POD1 and POD3, we captured the temporal pattern of postoperative inflammation (30). Unsupervised clustering identified three inflammatory phenotypes. After adjustment for 16 baseline variables, the delayed prolonged-risk phenotype, Cluster 3, remained strongly associated with AL (adjusted OR, 9.93; 95% CI, 4.69–21.04; *P <* 0.001). From a biological perspective, an early NLR increase after surgery may reflect an expected acute stress response. In Cluster 2, this response declined by POD3 and was not associated with increased AL risk. By contrast, persistent elevation at POD3 may indicate impaired inflammatory resolution (31). This sustained response could be linked to delayed epithelial repair, microvascular dysfunction, or ongoing local inflammatory injury at the anastomotic site (32). These mechanisms remain inferential, but they provide a plausible explanation for the incremental predictive value of the NLR trajectory in Model 2. This added value was supported by the IDI of 0.1157 and net NRI of 0.1289. Preoperative corrected serum calcium was another stable predictor selected by the ensemble strategy. Higher preoperative corrected calcium was independently associated with lower AL risk (adjusted OR, 0.20; 95% CI, 0.09–0.43; *P <* 0.001). This variable has received relatively limited attention in previous AL studies. Potential biological links include epithelial junction integrity, coagulation, smooth-muscle function, and tissue repair (33, 34). However, these pathways require further mechanistic validation. Postoperative corrected calcium did not remain independently associated with AL in Model 2 (adjusted OR, 0.69; 95% CI, 0.15–3.23; *P* = 0.636). This difference may reflect the greater influence of fluid resuscitation, acute-phase responses, and capillary leakage on postoperative calcium measurements. Preoperative corrected calcium may therefore better represent baseline physiological reserve than postoperative calcium values.The apparent protective associations of older age and alcohol consumption should be interpreted cautiously. These likely reflect residual confounding or selection bias — for example, surgeons may selectively offer sphincter-preserving procedures to fitter elderly patients — rather than true biological protection.

To support clinical translation, we deployed the models as a web-based dynamic nomogram (35). At the first decision point, immediately after resection, Model 1 can estimate baseline AL risk and inform early surveillance planning. At the second decision point, POD3, Model 2 can update risk using NLR trajectory and severe hypoalbuminaemia. This two-stage process may help identify patients who require closer examination, contrast evaluation, antimicrobial optimisation, or early surgical reassessment (36). Rather than replacing clinical judgement, the tool provides a structured way to update risk as perioperative information accumulates (24).

### 4.1 Limitations

This study has several limitations.First, the model was developed on a single-centre retrospective cohort, which may introduce selection bias and documentation variability. External validation in an independent multicentre population is necessary before clinical adoption (23). Second, the cohort (*N* = 815) provides narrow headroom above the Riley sample size threshold of 803 patients. We addressed this through LASSO shrinkage and 1,000 bootstrap resamples; however, computational constraints precluded repeating the full imputation and feature selection pipelines within each resample, and the reported optimism-corrected metrics may retain a degree of residual bias. Third, several potentially important predictors were unavailable. Intraoperative metrics such as indocyanine green (ICG) angiography, anastomotic perfusion assessment, surgeon volume, and stapler firing counts were not uniformly documented in the historical records. The model also lacks multimodal data — tissue molecular biomarkers, radiomics, and gut microbiota profiles — increasingly implicated in anastomotic healing (37, 38). Fourth, restricting postoperative monitoring to the first three days may miss delayed AL (39). Fifth, the apparent protective associations for older age and alcohol consumption should be interpreted cautiously, as they likely reflect residual confounding rather than true biological effects. Finally, although decision curve analysis demonstrated net benefit, prospective impact studies are needed to confirm whether model-guided intervention improves clinical outcomes.

## 5 Conclusions

This study developed and validated a longitudinal biomarker-informed two-stage sequential model for early AL risk prediction after rectal cancer surgery. The findings highlight the value of combining baseline physiological reserve, represented in part by preoperative corrected calcium, with postoperative inflammatory resolution patterns captured by longitudinal NLR trajectories. The sequential framework improved discrimination, calibration, and reclassification compared with the baseline model. Delivered through a web-based dynamic nomogram, this approach may support industrialized perioperative risk stratification and earlier targeted management, pending external prospective validation.

## Data Availability

The raw data supporting the conclusions of this article will be made available by the authors, without undue reservation.
