# Supplementary material for: A landmark-based dynamic prediction model for anastomotic leakage after rectal cancer surgery: integrating perioperative and postoperative trajectories
Source: Front Oncol. 2026 Sep 16;16:1915256. doi: 10.3389/fonc.2026.1915256 (PMC13623707; doi:10.3389/fonc.2026.1915256)
Supplement: Supplementary file 1 [file DataSheet1.pdf]

Supplementary Material

1 SUPPLEMENTARY TABLES

Table S1. Sensitivity analysis comparing the baseline risk model in the complete eligible cohort (N = 816) versus the primary landmark cohort (N = 815).

Variable	Sensitivity Cohort (N = 816)		Primary Cohort (N = 815)	
	OR (95% CI)	P Value	OR (95% CI)	P Value
**Independent Predictors ($P < 0.05$)**				
Sex (Male vs. Female)	4.27 (2.18–8.36)	< 0.001	4.64 (2.32–9.25)	< 0.001
Diverting stoma (Yes vs. No)	0.17 (0.08–0.34)	< 0.001	0.15 (0.07–0.32)	< 0.001
ASA grade (III–IV vs. I–II)	2.97 (1.62–5.45)	< 0.001	2.95 (1.60–5.45)	< 0.001
Preop corrected Ca (per mmol/L)	0.20 (0.09–0.43)	< 0.001	0.20 (0.09–0.43)	< 0.001
Distance from AV (per cm)	0.85 (0.78–0.93)	< 0.001	0.85 (0.78–0.93)	< 0.001
Prior abdominal surgery (Yes vs. No)	2.50 (1.39–4.52)	0.002	2.35 (1.29–4.28)	0.005
Age (per year)	0.97 (0.94–0.99)	0.017	0.96 (0.94–0.99)	0.011
Alcohol consumption (Yes vs. No)	0.41 (0.19–0.85)	0.018	0.40 (0.19–0.85)	0.017
Preop Hb (per g/L)	0.98 (0.97–1.00)	0.029	0.99 (0.97–1.00)	0.043
Operative time (per min)	1.00 (1.00–1.01)	0.030	1.00 (1.00–1.01)	0.049
**Non-independent Predictors ($P \geq 0.05$)**				
Hypertension (Yes vs. No)	1.71 (0.96–3.02)	0.067	1.77 (0.99–3.14)	0.053
NAC (Yes vs. No)	1.83 (0.94–3.56)	0.075	1.88 (0.96–3.67)	0.065
Clinical stage				
Stage 2 (vs. Stage 1)	1.08 (0.09–12.87)	0.954	1.10 (0.09–13.39)	0.940
Stage 3 (vs. Stage 1)	3.72 (0.35–39.06)	0.274	3.79 (0.35–40.57)	0.271
Stage 4 (vs. Stage 1)	2.82 (0.24–33.68)	0.413	2.91 (0.24–35.42)	0.403
Tumor size (per cm)	1.05 (0.89–1.25)	0.544	1.05 (0.89–1.25)	0.562
CEA (per ng/mL)	1.00 (0.99–1.00)	0.630	1.00 (0.99–1.00)	0.626
EBL (per mL)	1.00 (1.00–1.00)	0.793	1.00 (1.00–1.00)	0.829

Note: ASA, American Society of Anesthesiologists; AV, anal verge; Ca, calcium; CEA, carcinoembryonic antigen; CI, confidence interval; EBL, estimated blood loss; Hb, hemoglobin; NAC, neoadjuvant chemotherapy; OR, odds ratio; Preop, preoperative.

This table presents a head-to-head sensitivity analysis to address potential selection bias. The **Sensitivity Cohort** (N = 816) represents the complete eligible surgical cohort, including the single patient diagnosed with early anastomotic leakage within 3 postoperative days. The **Primary Cohort** (N = 815) represents the landmark cohort utilized for sequential dynamic modeling in the main manuscript. The virtually identical point estimates and statistical significance thresholds across all 16 consensus predictors demonstrate that the cohort restriction introduced no selection bias. ($P < 0.05$ indicates statistical significance)

Table S2. PAM Clustering Parameters and Assignment Rules for Future Patients

Parameter	Inflammatory Residue ($X_{\mathbf{POD3}}$)	Acute Surgical Surge ($Y_{\mathbf{POD1}}$)
Scaling Parameters		
Mean (μ)	4.980	9.469
Standard Deviation (σ)	5.389	7.338
Cluster Medoids (Standardized Coordinates)		
Medoid: Cluster 1 (Safe)	-0.170	0.754
Medoid: Cluster 2 (Surge)	-0.417	-0.661
Medoid: Cluster 3 (Danger)	1.771	0.302

Mathematical Rule for Assigning Future Patients to NLR Trajectory Clusters:
To classify a prospective patient into one of the three established clusters, the following three steps must be performed:
Step 1: Feature Calculation. Calculate the raw dynamic inflammatory features based on the prospective patient's NLR values:

- Acute Surgical Surge (Y_{new}) = POD1 NLR $-$ Baseline NLR
- Inflammatory Residue (X_{new}) = POD3 NLR $-$ Baseline NLR

Step 2: Standardization. Standardize the raw features using the exact scaling parameters (Mean μ and Standard Deviation σ) derived from this training cohort:

- $Y_{\text{scaled}} = (Y_{\text{new}} - 9.469)/7.338$
- $X_{\text{scaled}} = (X_{\text{new}} - 4.980)/5.389$

Step 3: Minimum Euclidean Distance Assignment. Calculate the Euclidean distance (d) between the patient's standardized coordinate ($X_{\text{scaled}}, Y_{\text{scaled}}$) and each of the three prespecified Cluster Medoids (M_1, M_2, M_3) provided in the table above:

- $d_i = \sqrt{(X_{\text{scaled}} - X_{M_i})^2 + (Y_{\text{scaled}} - Y_{M_i})^2}$, for $i \in \{1, 2, 3\}$

The prospective patient is then categorically assigned to the cluster i that yields the minimum distance (d_i).

Table S3. Comparison of discrimination and calibration performance across machine learning algorithms and clinical prediction models.

Methods	Brier Score	MAE (%)	$\text{E}_{\max}$ (%)	AUC (95% CI)
Stepwise AIC	0.0659	2.41	18.48	0.824 (95% CI: 0.774–0.874)
SVM-RFE	0.0738	1.02	13.17	0.812 (95% CI: 0.763–0.862)
RF-MDA	0.0706	1.62	11.84	0.813 (95% CI: 0.761–0.866)
LASSO	0.0651	2.53	19.63	0.824 (95% CI: 0.773–0.875)
Model 1	0.0657	2.28	17.64	0.825 (95% CI: 0.774–0.875)
Model 2	0.0557	1.14	6.82	0.889 (95% CI: 0.848–0.930)

Note: AUC, area under the receiver operating characteristic curve; CI, confidence interval; MAE, mean absolute error; $E_{\max}$, maximum absolute error; LASSO, least absolute shrinkage and selection operator; RF, random forest; MDA, mean decrease accuracy; SVM, support vector machine; RFE, recursive feature elimination.

Table S4. Reclassification and Discrimination Improvement Metrics After Incorporating Postoperative Dynamic Indicators into the Baseline Model

Metric	Estimate (95% CI)	*P* Value
Integrated Discrimination Improvement (IDI)	0.1157 (95% CI: 0.0760–0.1616)	< 0.001
Net Reclassification Improvement (NRI)[a]	0.1289 (95% CI: 0.0404–0.2179)	0.006

Note: CI, confidence interval; IDI, integrated discrimination improvement; NRI, net reclassification improvement.

[a] The Net Reclassification Improvement (NRI) was calculated using a prespecified clinical risk threshold (cutoff) of 10% to capture the shift in risk stratification for anastomotic leakage. All statistical evaluations were two-sided, and a *P* value < 0.05 was considered statistically significant.

Table S5. Full model specification of the baseline multivariable logistic regression analysis for anastomotic leakage (Model 1).

Variable	Coefficient (β)	OR (95% CI)	*P* Value
**Independent Predictors (*P* < 0.05)**			
Sex (Male vs. Female)	1.5347	4.64 (2.32–9.25)	< 0.001
Diverting stoma (Yes vs. No)	-1.8971	0.15 (0.07–0.32)	< 0.001
ASA grade (III-IV vs. I-II)	1.0818	2.95 (1.60–5.45)	< 0.001
Preop corrected Ca (per mmol/L)	-1.6094	0.20 (0.09–0.43)	< 0.001
Distance from AV (per cm)	-0.1625	0.85 (0.78–0.93)	< 0.001
Prior abdominal surgery (Yes vs. No)	0.8544	2.35 (1.29–4.28)	0.005
Age (per year)	-0.0408	0.96 (0.94–0.99)	0.011
Alcohol consumption (Yes vs. No)	-0.9163	0.40 (0.19–0.85)	0.017
Preop Hb (per g/L)	-0.0101	0.99 (0.97–1.00)	0.043
Operative time (per min)	0.0000	1.00 (1.00–1.01)	0.049
**Non-independent Predictors (*P* ≥ 0.05)**			
Hypertension (Yes vs. No)	0.5710	1.77 (0.99–3.14)	0.053
NAC (Yes vs. No)	0.6313	1.88 (0.96–3.67)	0.065
Clinical stage			
Stage 2 (vs. Stage 1)	0.0953	1.10 (0.09–13.39)	0.940
Stage 3 (vs. Stage 1)	1.3324	3.79 (0.35–40.57)	0.271
Stage 4 (vs. Stage 1)	1.0682	2.91 (0.24–35.42)	0.403
Tumor size (per cm)	0.0488	1.05 (0.89–1.25)	0.562
CEA (per ng/mL)	0.0000	1.00 (0.99–1.00)	0.626
EBL (per mL)	0.0000	1.00 (1.00–1.00)	0.829
**Intercept (Constant β_0)**	**2.8087**	–	–

Note: ASA, American Society of Anesthesiologists; AV, anal verge; Ca, calcium; CEA, carcinoembryonic antigen; CI, confidence interval; EBL, estimated blood loss; Hb, hemoglobin; NAC, neoadjuvant chemotherapy; OR, odds ratio; Preop, preoperative.

Variables are ordered hierarchically by their statistical significance within the baseline multivariable model. The regression coefficient (β) and model intercept are explicitly provided to allow for independent mathematical calculation of individualized risk probabilities. Categorical variables with multiple levels (Clinical stage) are evaluated simultaneously using multi-degree-of-freedom tests against their designated reference category (Stage 1). (*P* < 0.05 indicates statistical significance).

Table S6. Full model specification of the enhanced dynamic multivariable logistic regression analysis for anastomotic leakage (Model 2).

Variable	Coefficient (β)	OR (95% CI)	*P* Value
**Independent Predictors (*P* < 0.05)**			
NLR trajectory cluster			
Cluster 3 (vs. Cluster 1)	2.2956	9.93 (4.69–21.04)	< 0.001
Sex (Male vs. Female)	1.7984	6.04 (2.80–13.00)	< 0.001
Postop ALB ($<$ 30 g/L vs. $\geq$ 30 g/L)	1.6525	5.22 (2.36–11.54)	< 0.001
Diverting stoma (Yes vs. No)	-2.4079	0.09 (0.04–0.22)	< 0.001
ASA grade (III-IV vs. I-II)	1.1600	3.19 (1.60–6.36)	< 0.001
Preop corrected Ca (per mmol/L)	-1.9661	0.14 (0.06–0.34)	< 0.001
Distance from AV (per cm)	-0.1508	0.86 (0.78–0.95)	0.002
Age (per year)	-0.0408	0.96 (0.93–0.99)	0.004
Prior abdominal surgery (Yes vs. No)	0.9083	2.48 (1.28–4.79)	0.007
Alcohol consumption (Yes vs. No)	-1.0217	0.36 (0.16–0.81)	0.013
NAC (Yes vs. No)	0.9123	2.49 (1.17–5.28)	0.018
Hypertension (Yes vs. No)	0.6729	1.96 (1.03–3.73)	0.041
**Non-independent Predictors (*P* ≥ 0.05)**			
Operative time (per min)	0.0000	1.00 (1.00–1.01)	0.069
Preop Hb (per g/L)	-0.0101	0.99 (0.97–1.00)	0.179
Clinical stage			
Stage 2 (vs. Stage 1)	-0.1744	0.84 (0.06–10.88)	0.891
Stage 3 (vs. Stage 1)	1.4929	4.45 (0.40–49.51)	0.225
Stage 4 (vs. Stage 1)	1.2442	3.47 (0.27–44.84)	0.341
CEA (per ng/mL)	-0.0101	0.99 (0.99–1.00)	0.257
Tumor size (per cm)	0.0488	1.05 (0.87–1.28)	0.604
Postop corrected Ca (per mmol/L)	-0.3711	0.69 (0.15–3.23)	0.636
EBL (per mL)	0.0000	1.00 (1.00–1.00)	0.933
NLR trajectory cluster			
Cluster 2 (vs. Cluster 1)	0.0100	1.01 (0.46–2.21)	0.982
**Intercept (Constant β_0)**	**2.5571**	**–**	**–**

Note: ALB, albumin; ASA, American Society of Anesthesiologists; AV, anal verge; Ca, calcium; CEA, carcinoembryonic antigen; CI, confidence interval; EBL, estimated blood loss; Hb, hemoglobin; NAC, neoadjuvant chemotherapy; NLR, neutrophil-to-lymphocyte ratio; OR, odds ratio; POD, postoperative day; Postop, postoperative; Preop, preoperative.

Variables are ordered hierarchically by their statistical significance within the multivariable enhanced model. Model 2 evaluates the incremental predictive value of early postoperative parameters after adjusting for all 16 baseline consensus predictors from Model 1. The regression coefficient (β) and model intercept are explicitly provided to allow for independent mathematical calculation of individualized risk probabilities. Categorical variables with multiple levels (Clinical stage and NLR trajectory cluster) are evaluated simultaneously using multi-degree-of-freedom tests against their designated reference categories (Stage 1 and Cluster 1, respectively). ($P < 0.05$ indicates statistical significance).

1.1 Detailed Eligibility Criteria

The detailed eligibility criteria were predefined before cohort construction. Eligible patients were adults ($\geq$18 years) with histologically confirmed rectal adenocarcinoma who underwent elective curative-intent rectal resection with primary colorectal anastomosis.

Patients were excluded based on the following criteria: (1) emergency surgery due to acute complications, including obstruction, perforation, or uncontrolled bleeding; (2) multiple primary colorectal malignancies; (3) concomitant rectovaginal or rectourethral fistulas; (4) missing key clinical, laboratory, operative, or postoperative data required for model development; (5) active systemic infection before surgery; (6) severe cardiopulmonary comorbidities that could substantially influence perioperative management; and (7) extreme physiological abnormalities considered non-representative of the target surgical population.

Severe cardiopulmonary comorbidities were defined as New York Heart Association (NYHA) class III–IV heart failure, myocardial infarction within 6 months before surgery, or severe chronic obstructive pulmonary disease requiring long-term home oxygen therapy. Extreme physiological abnormalities were defined as body mass index (BMI) < 15 or > 40 kg/m^2, or baseline serum albumin concentration < 15 g/L.

After applying these criteria, 816 patients were included in the final analytical cohort.

2 SUPPLEMENTARY FIGURES

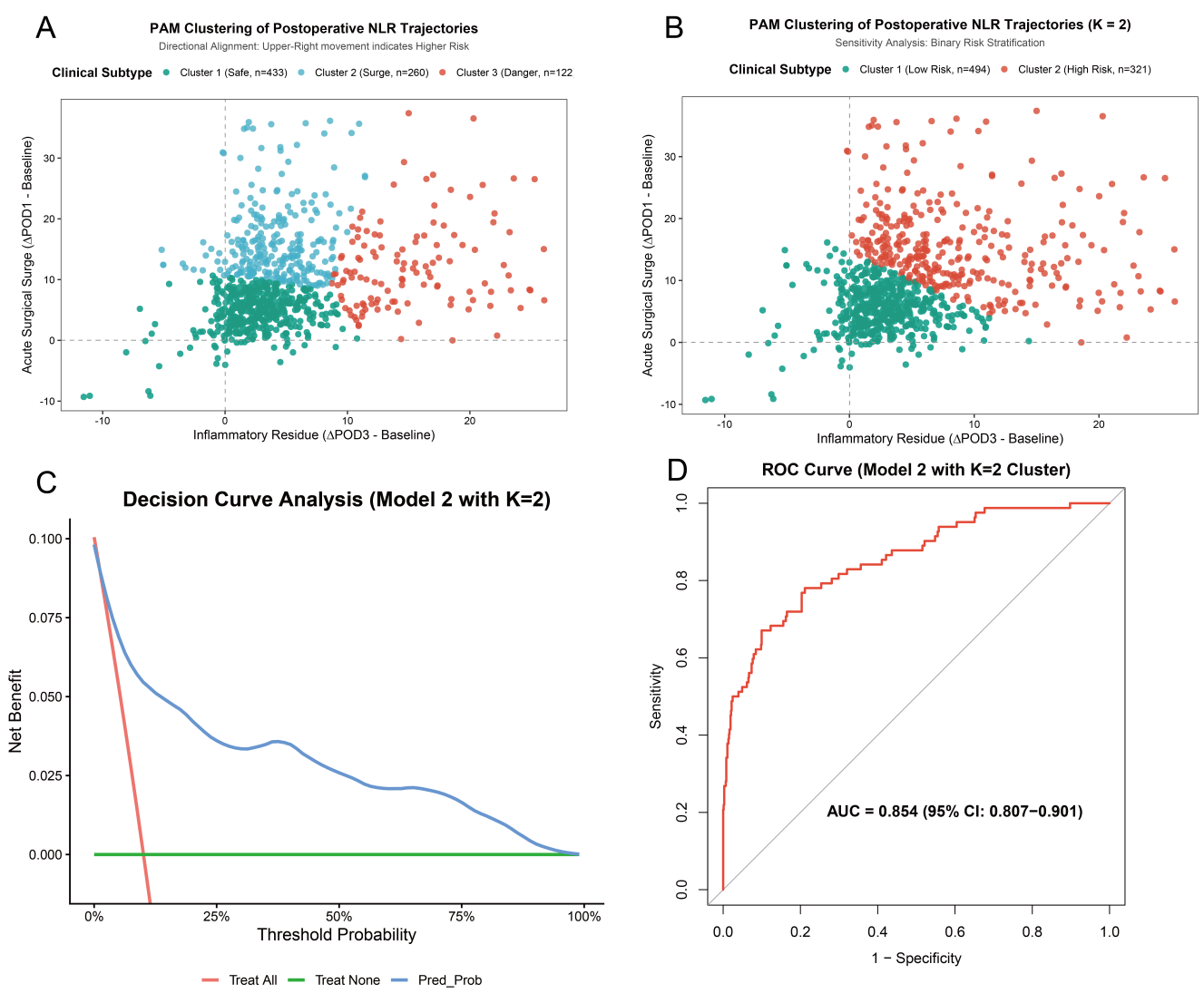

Figure S1. Supplementary Figure S1. Postoperative NLR trajectory clustering and performance evaluation of the alternative binary model. **(A)** Scatter plot of PAM clustering ($K = 3$) based on early dynamic changes. Patients are stratified into three distinct clinical subtypes: Cluster 1 (Safe, $n = 433$, green), Cluster 2 (Surge, $n = 260$, blue), and Cluster 3 (Danger, $n = 122$, red). The x-axis represents the inflammatory residue (ΔPOD3 minus Baseline), and the y-axis represents the acute surgical surge (ΔPOD1 minus Baseline). **(B)** Sensitivity analysis demonstrating binary risk stratification (PAM clustering, $K = 2$). Patients are divided into Cluster 1 (Low Risk, $n = 494$, green) and Cluster 2 (High Risk, $n = 321$, red). **(C)** Decision curve analysis (DCA) evaluating the net benefit of Model 2 when incorporating the alternative binary ($K = 2$) cluster variable. **(D)** Receiver operating characteristic (ROC) curve for Model 2 with the $K = 2$ cluster variable, yielding an AUC of 0.854 (95% CI: 0.807–0.901). **Note:** NLR, neutrophil-to-lymphocyte ratio; POD1, postoperative day 1; POD3, postoperative day 3; PAM, partitioning around medoids; AUC, area under the curve; CI, confidence interval.

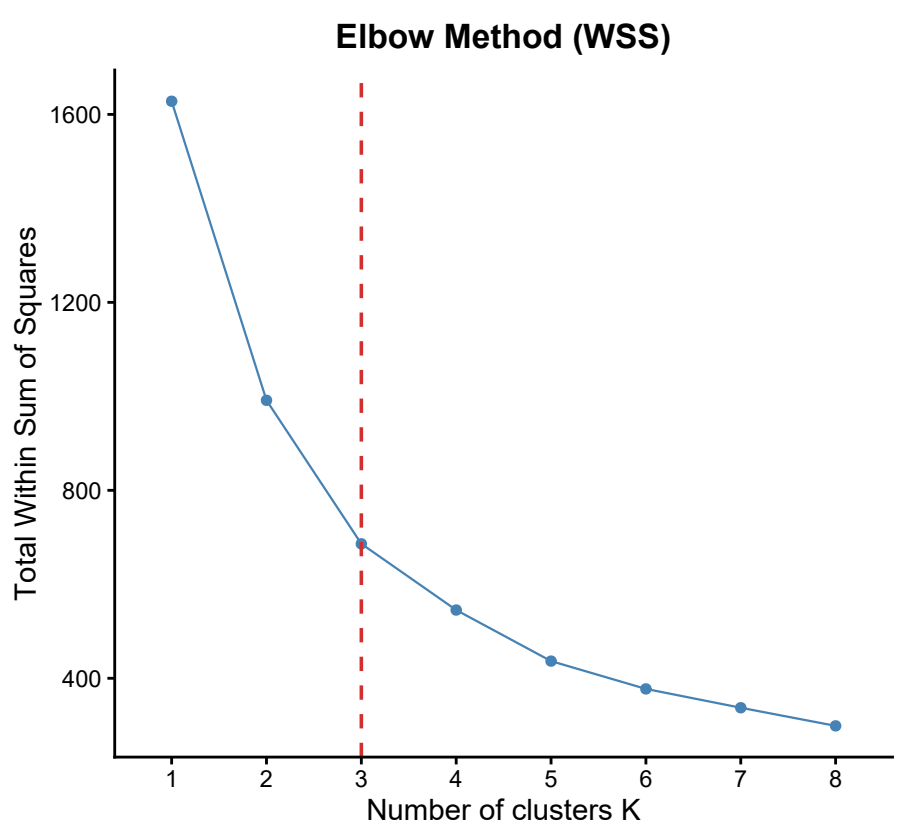

Figure S2. Determination of the optimal number of clusters using the elbow method. The line graph displays the total within sum of squares against the number of clusters (K). The red dashed line indicates $K = 3$ as the optimal inflection point (the ”elbow”), where the rate of variance drop significantly slows down.

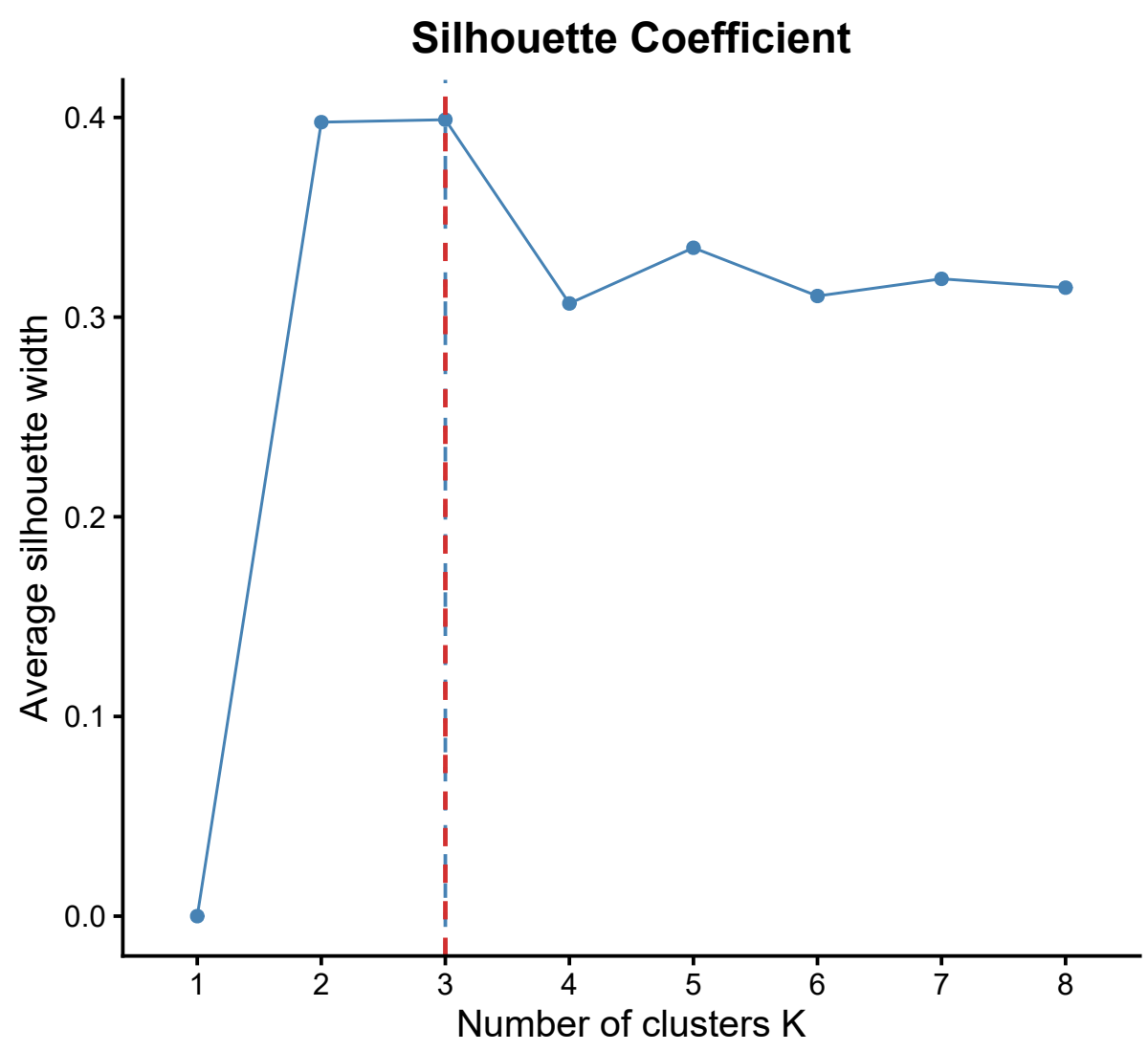

Figure S3. Evaluation of clustering quality using the silhouette coefficient. The plot illustrates the average silhouette width across different numbers of clusters (K). The red dashed line highlights $K = 3$ as the optimal choice, which yields the highest average silhouette width, confirming robust clustering separation and cohesion.

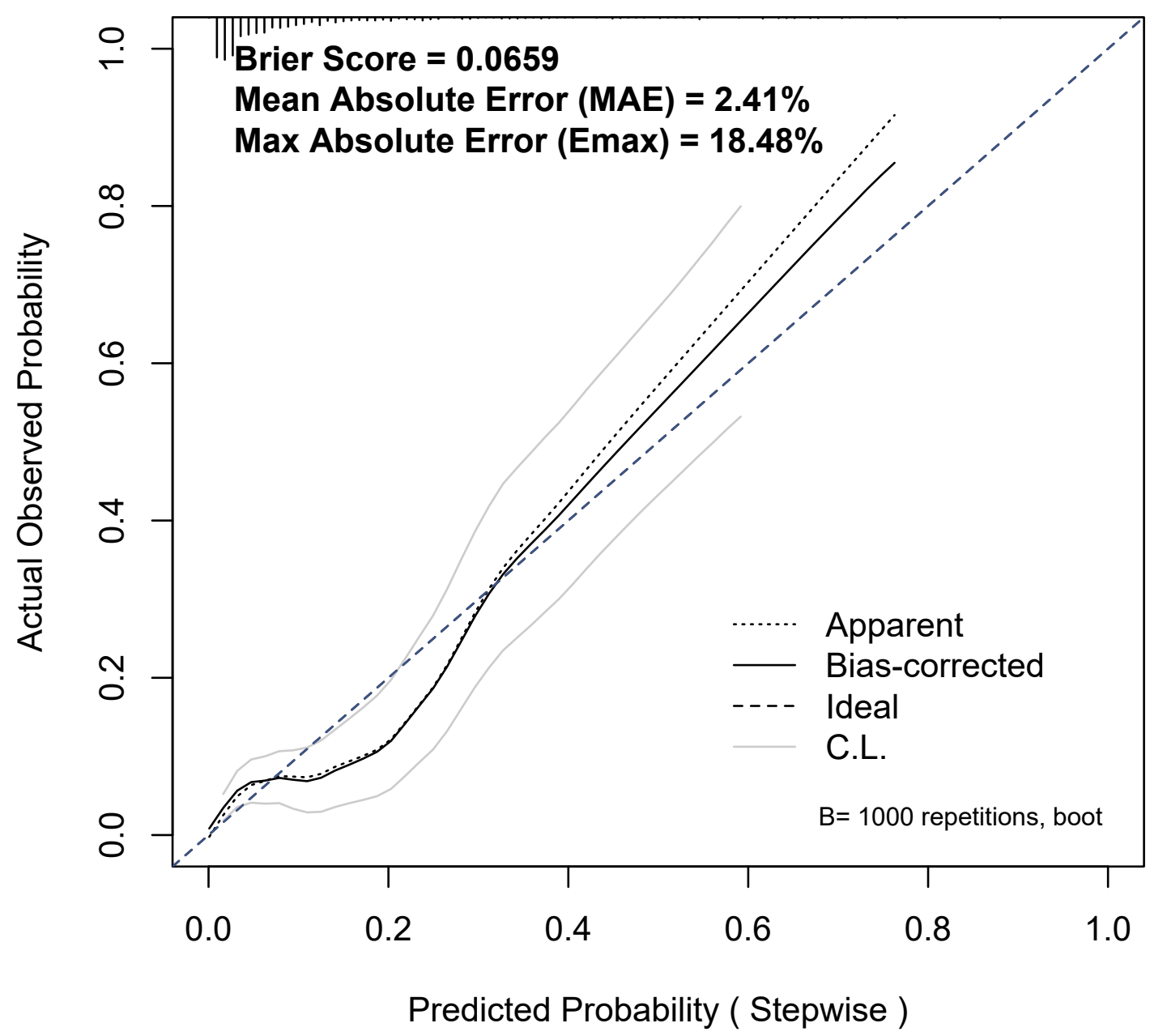

Figure S4. Calibration curve of the stepwise logistic regression model internally validated by 1,000 bootstrap resamples. The x-axis represents the predicted probability, and the y-axis represents the actual observed probability. The dashed diagonal line indicates perfect prediction (Ideal). The dotted line represents the apparent predictive accuracy (Apparent), while the solid black line indicates the bias-corrected calibration (Bias-corrected). The lighter solid lines outline the 95% confidence limits (C.L.). Overall model performance is summarized by a Brier Score of 0.0659, a Mean Absolute Error (MAE) of 2.41%, and a Maximum Absolute Error ($E_{\max}$) of 18.48%.

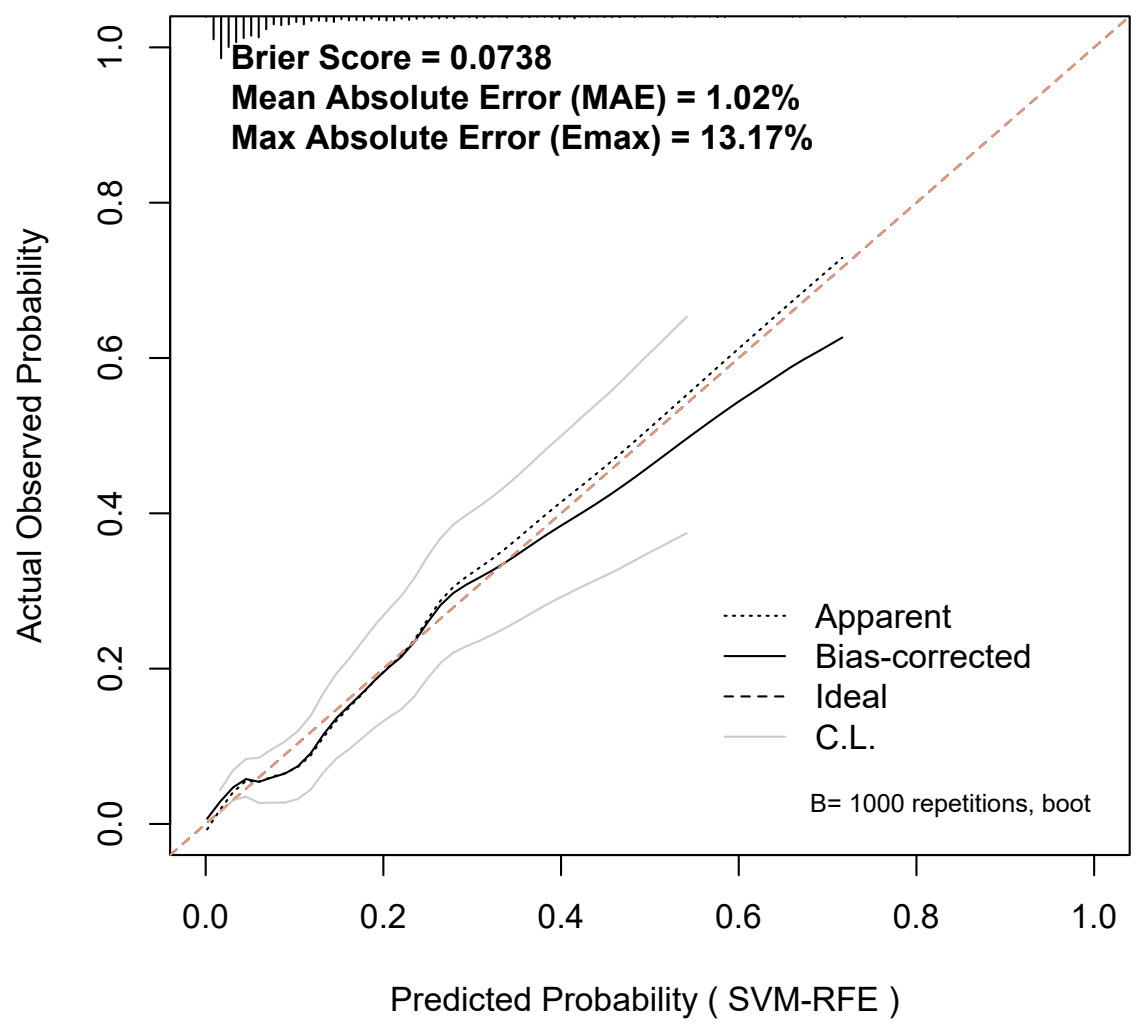

Figure S5. Calibration curve of the support vector machine (SVM-RFE) model internally validated by 1,000 bootstrap resamples. The x-axis represents the predicted probability, and the y-axis represents the actual observed probability. The dashed diagonal line indicates perfect prediction (Ideal). The dotted line represents the apparent predictive accuracy (Apparent), while the solid black line indicates the bias-corrected calibration (Bias-corrected). The lighter solid lines outline the 95% confidence limits (C.L.). Overall model performance is summarized by a Brier Score of 0.0738, a Mean Absolute Error (MAE) of 1.02%, and a Maximum Absolute Error ($E_{\max}$) of 13.17%.

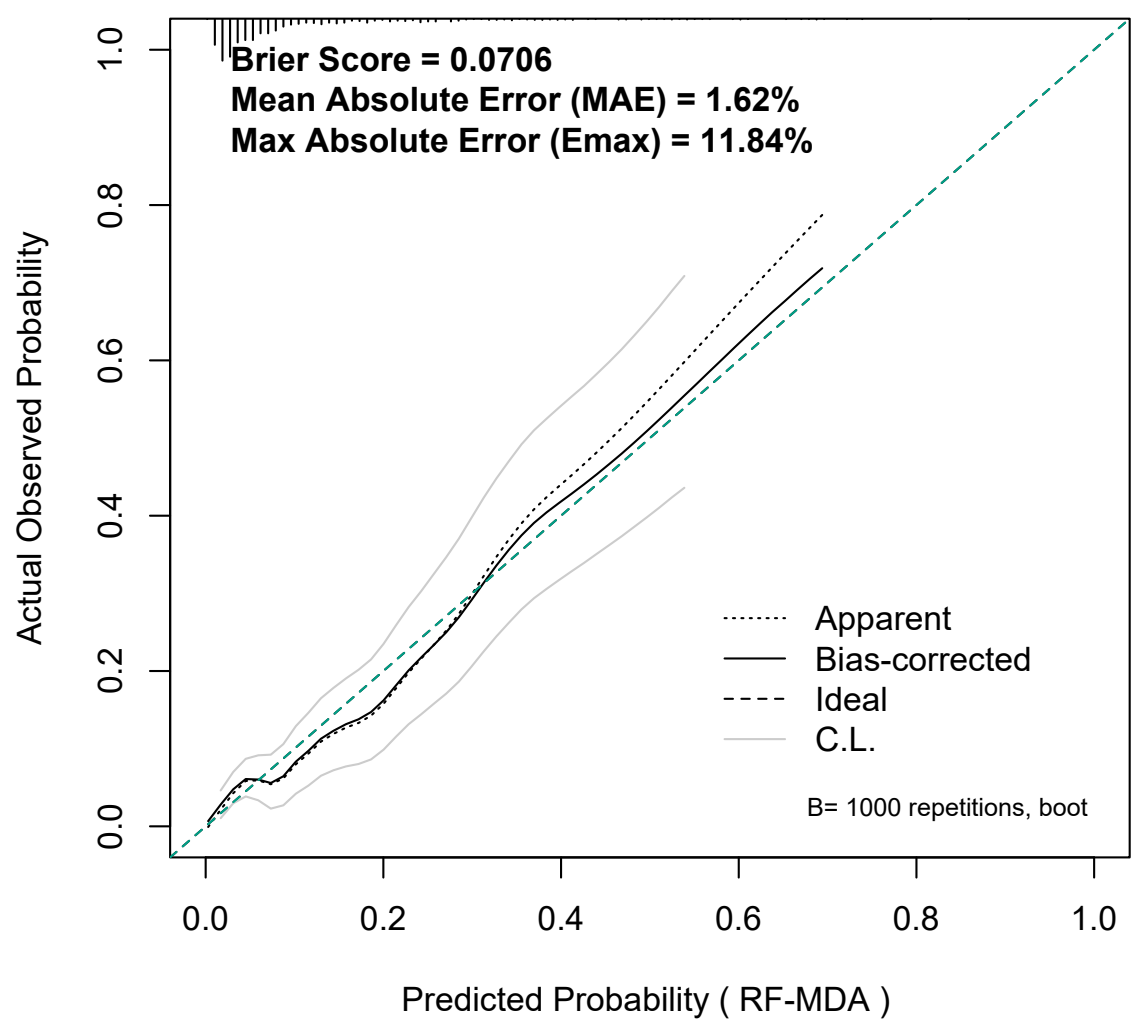

Figure S6. Calibration curve of the random forest (RF-MDA) model internally validated by 1,000 bootstrap resamples. The x-axis represents the predicted probability, and the y-axis represents the actual observed probability. The dashed diagonal line indicates perfect prediction (Ideal). The dotted line represents the apparent predictive accuracy (Apparent), while the solid black line indicates the bias-corrected calibration (Bias-corrected). The lighter solid lines outline the 95% confidence limits (C.L.). Overall model performance is summarized by a Brier Score of 0.0706, a Mean Absolute Error (MAE) of 1.62%, and a Maximum Absolute Error ($E_{\max}$) of 11.84%.

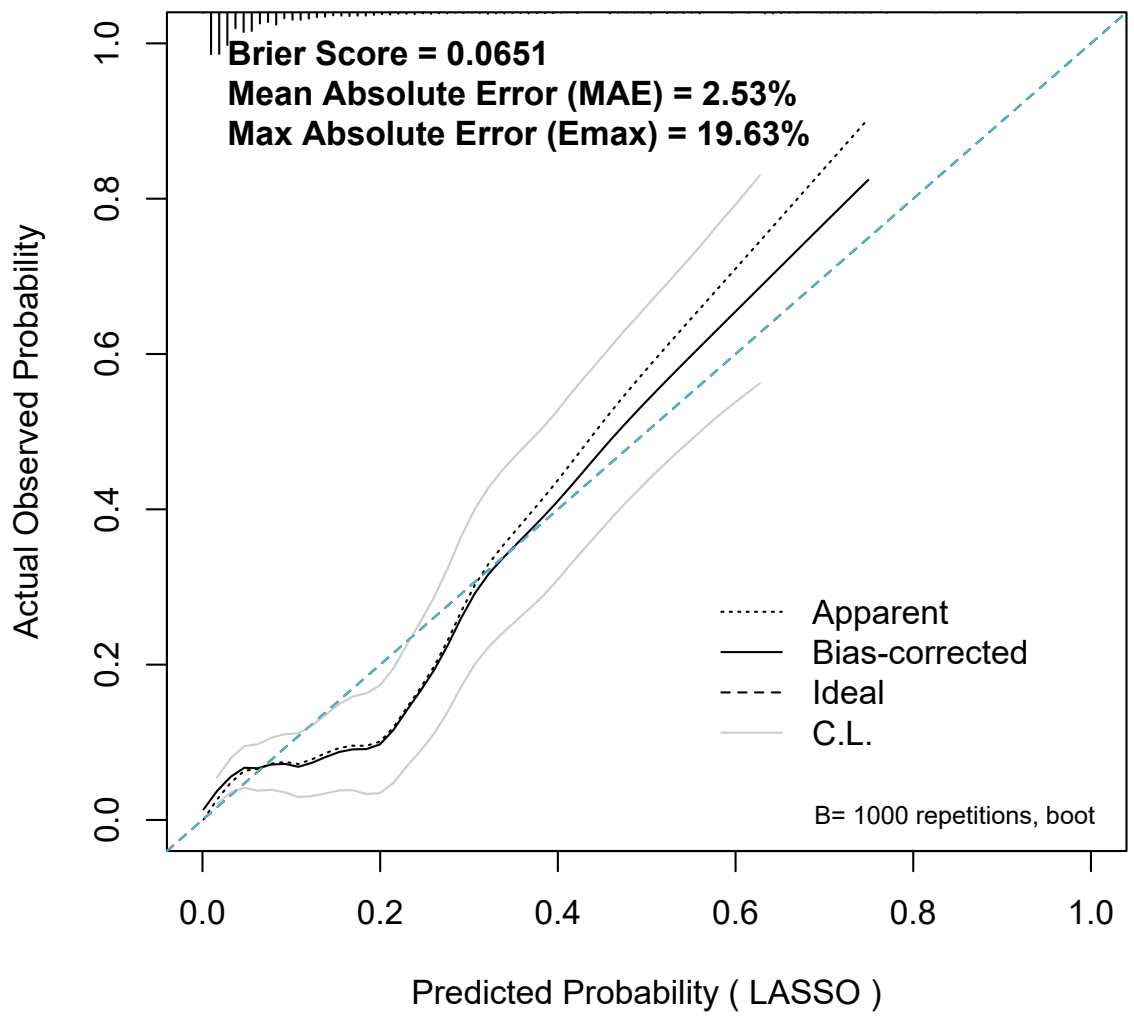

Figure S7. Calibration curve of the LASSO regression model internally validated by 1,000 bootstrap resamples. The x-axis represents the predicted probability, and the y-axis represents the actual observed probability. The dashed diagonal line indicates perfect prediction (Ideal). The dotted line represents the apparent predictive accuracy (Apparent), while the solid black line indicates the bias-corrected calibration (Bias-corrected). The lighter solid lines outline the 95% confidence limits (C.L.). Overall model performance is summarized by a Brier Score of 0.0651, a Mean Absolute Error (MAE) of 2.53%, and a Maximum Absolute Error ($E_{\max}$) of 19.63%.

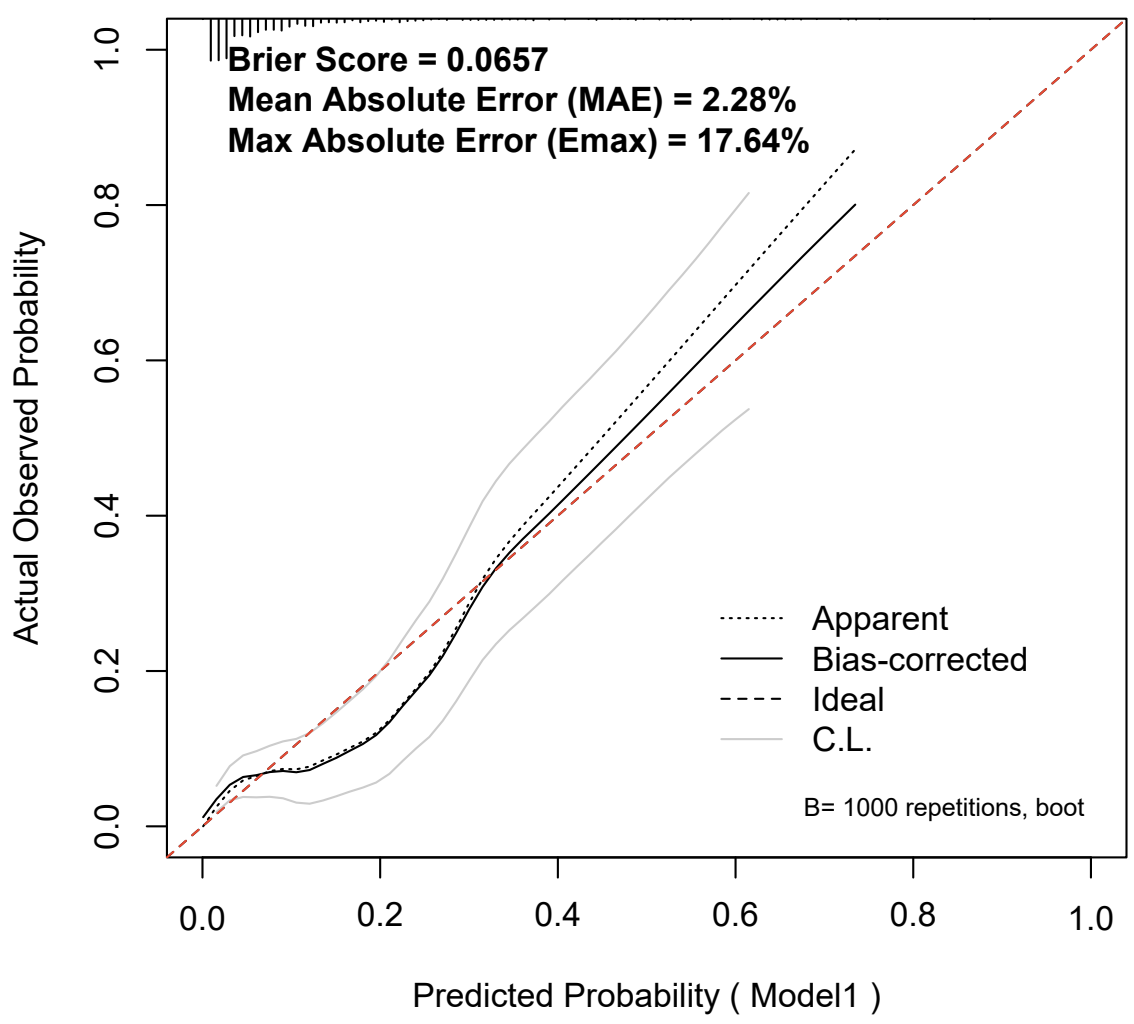

Figure S8. Calibration curve of the preoperative baseline model (Model 1) internally validated by 1,000 bootstrap resamples. The x-axis represents the predicted probability, and the y-axis represents the actual observed probability. The dashed diagonal line indicates perfect prediction (Ideal). The dotted line represents the apparent predictive accuracy (Apparent), while the solid black line indicates the bias-corrected calibration (Bias-corrected). The lighter solid lines outline the 95% confidence limits (C.L.). Overall model performance is summarized by a Brier Score of 0.0657, a Mean Absolute Error (MAE) of 2.28%, and a Maximum Absolute Error ($E_{\max}$) of 17.64%.

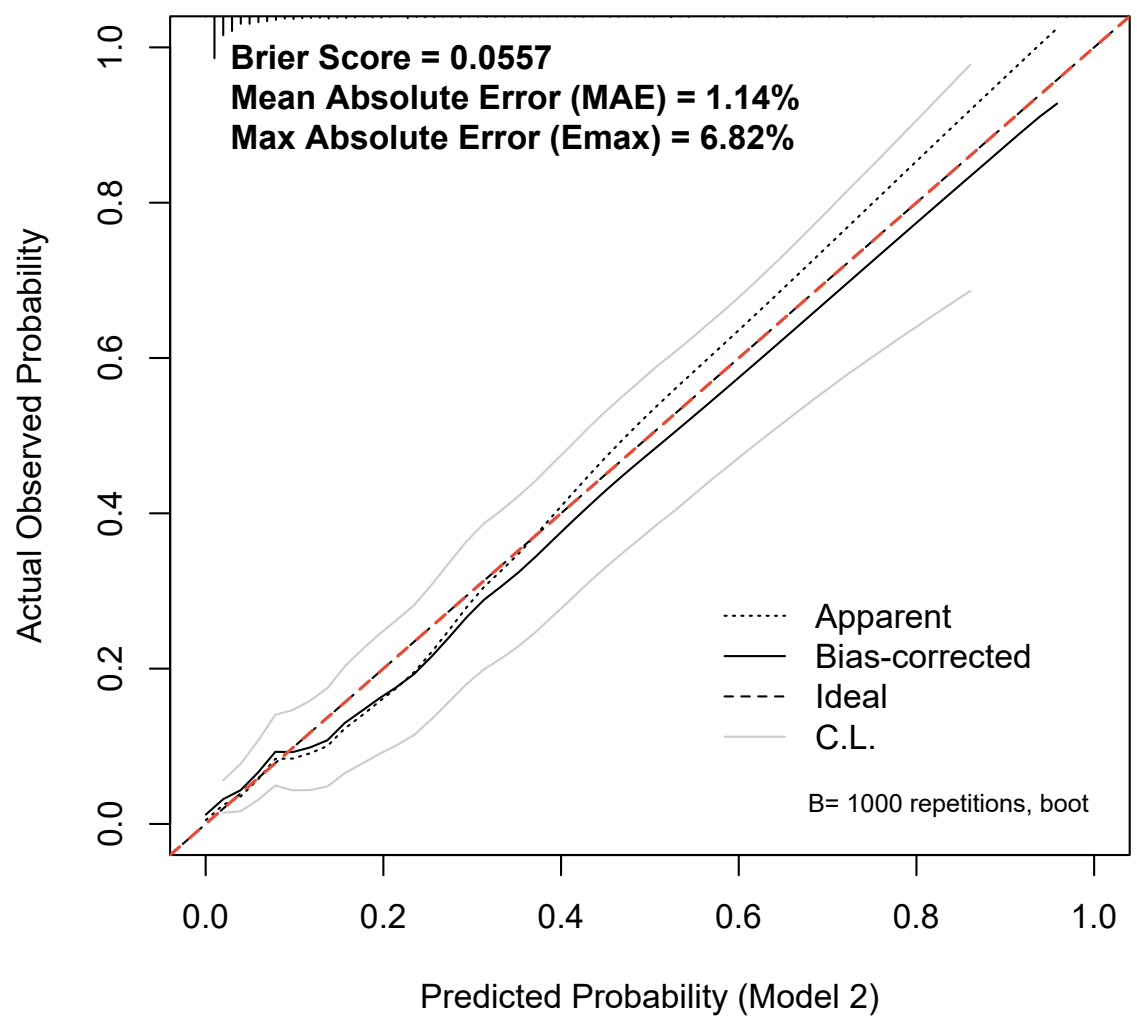

Figure S9. Calibration curve of the postoperative enhanced model (Model 2) internally validated by 1,000 bootstrap resamples. The x-axis represents the predicted probability, and the y-axis represents the actual observed probability. The dashed diagonal line indicates perfect prediction (Ideal). The dotted line represents the apparent predictive accuracy (Apparent), while the solid black line indicates the bias-corrected calibration (Bias-corrected). The lighter solid lines outline the 95% confidence limits (C.L.). Overall model performance is summarized by a Brier Score of 0.0557, a Mean Absolute Error (MAE) of 1.14%, and a Maximum Absolute Error (E_{max}) of 6.82%.

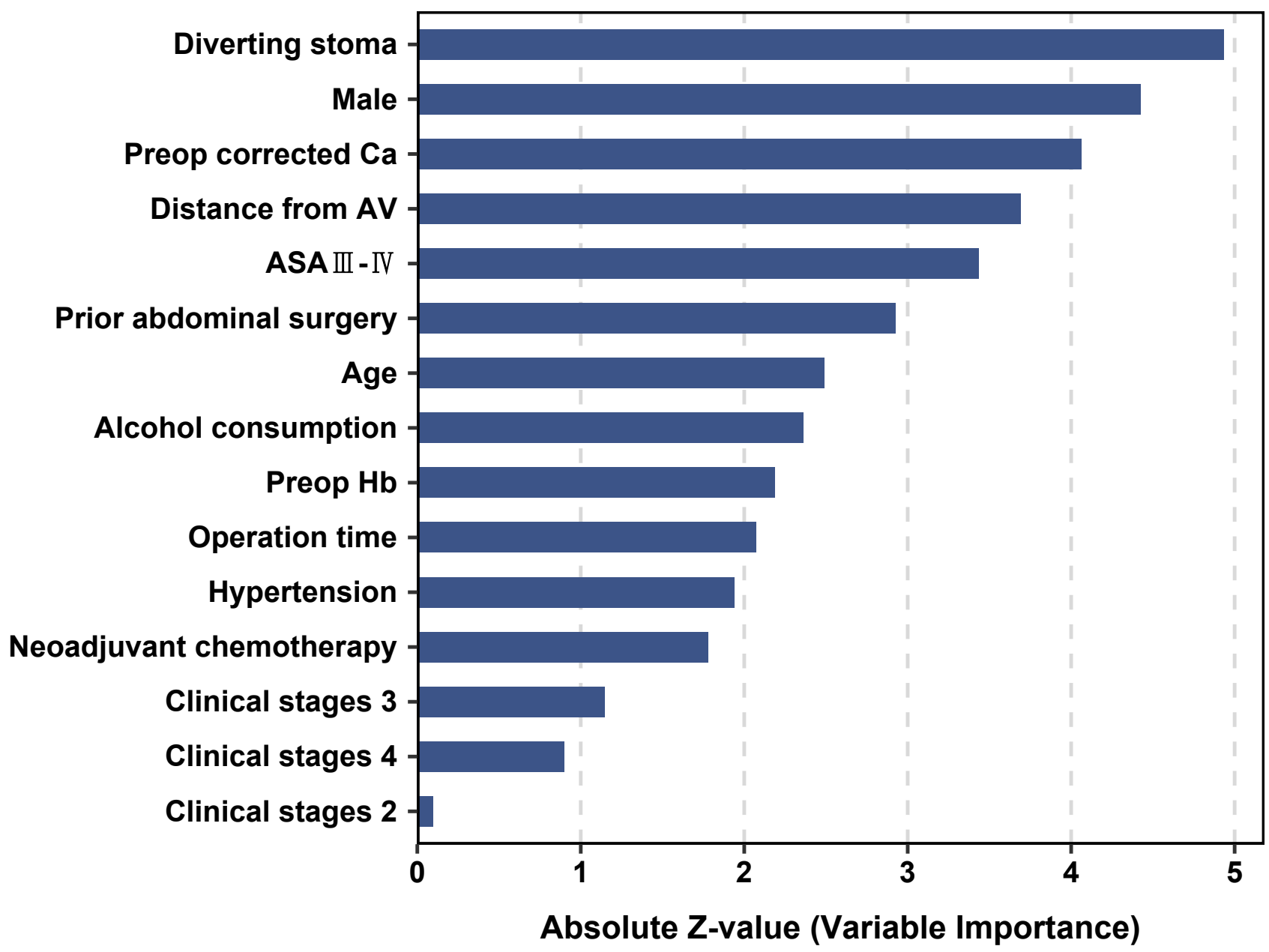

Figure S10. Variable importance measured by the absolute Z-statistic in the stepwise logistic regression. The horizontal bar chart illustrates the relative predictive power of each clinical feature. Variables are ranked in descending order according to their absolute Z-values, with ”Diverting stoma”, ”Male” (Sex), and ”Preop corrected Ca” emerging as the top three most influential predictors in the stepwise model.

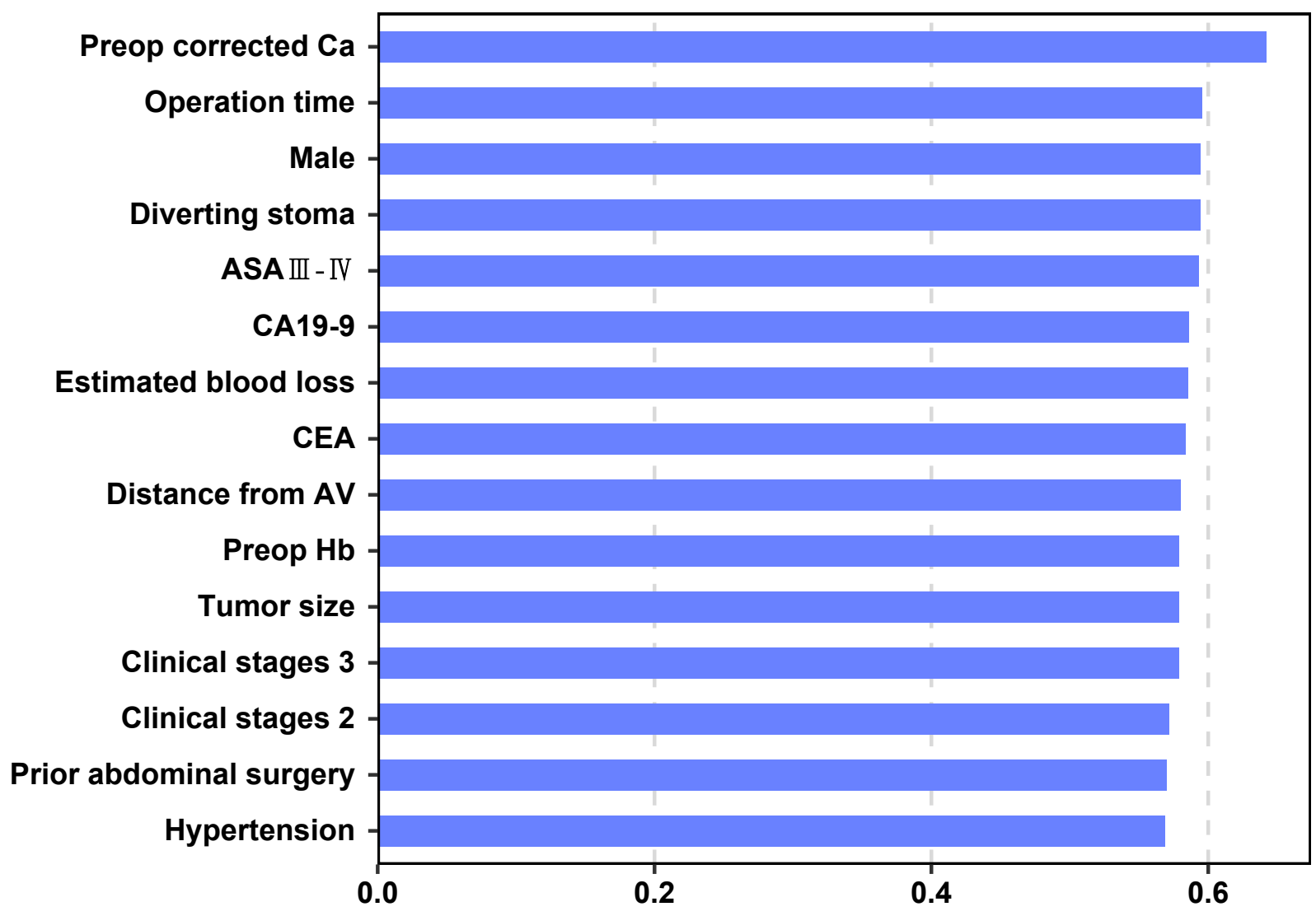

Figure S11. Variable importance evaluation derived from the support vector machine (SVM-RFE) model. The horizontal bar chart displays the relative weights assigned to different baseline clinical variables during the recursive feature elimination process. ”Preop corrected Ca”, ”Operation time”, and ”Male” (Sex) represent the features that contribute most significantly to the SVM classification boundary.

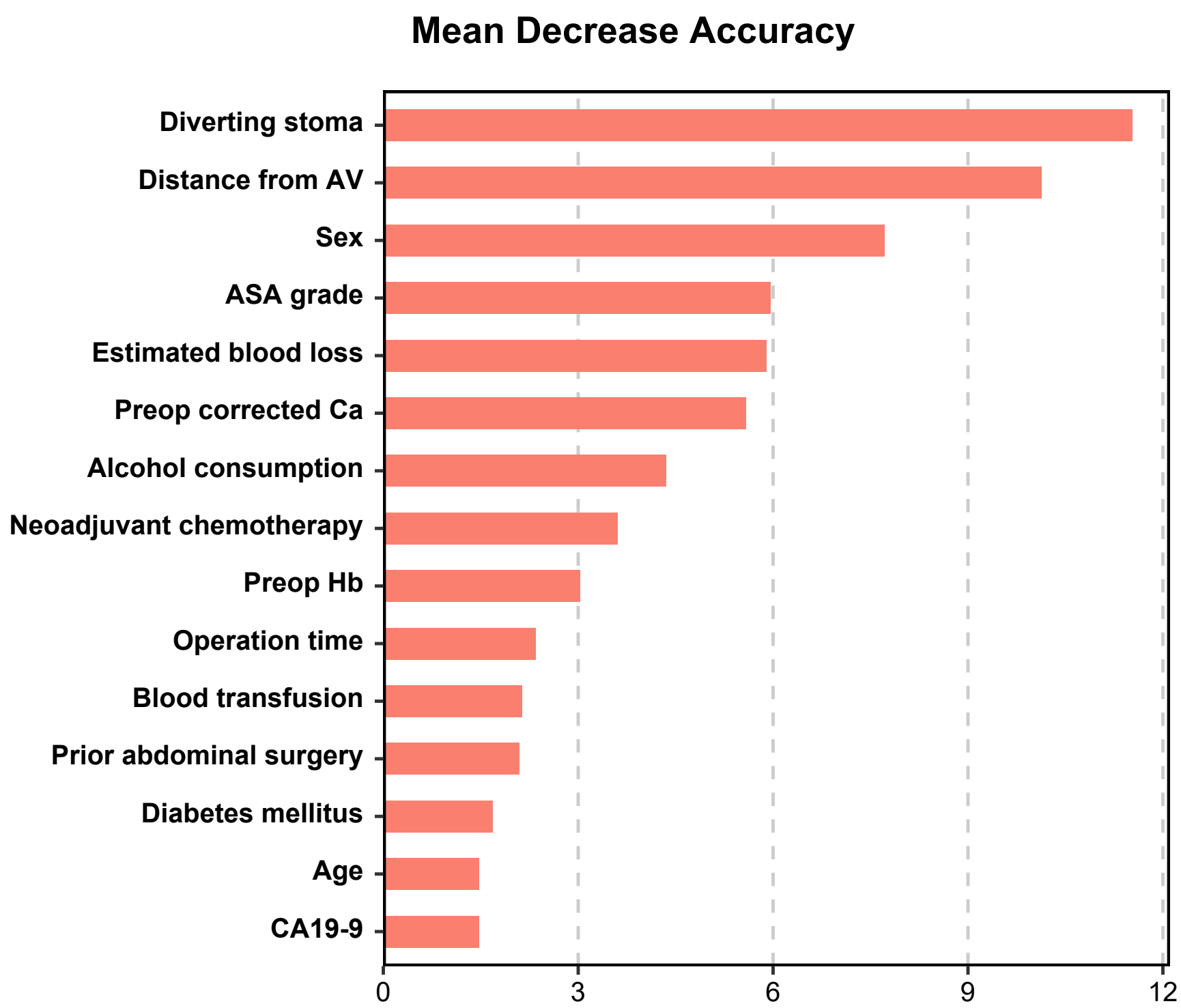

Figure S12. Variable importance ranking based on the mean decrease accuracy (MDA) of the random forest algorithm. The chart demonstrates how much the model’s accuracy decreases when a specific variable is excluded or permuted. A higher MDA value indicates greater importance for accurate classification. ”Diverting stoma” and ”Distance from AV” are identified as the most critical nodes for decision tree splits.

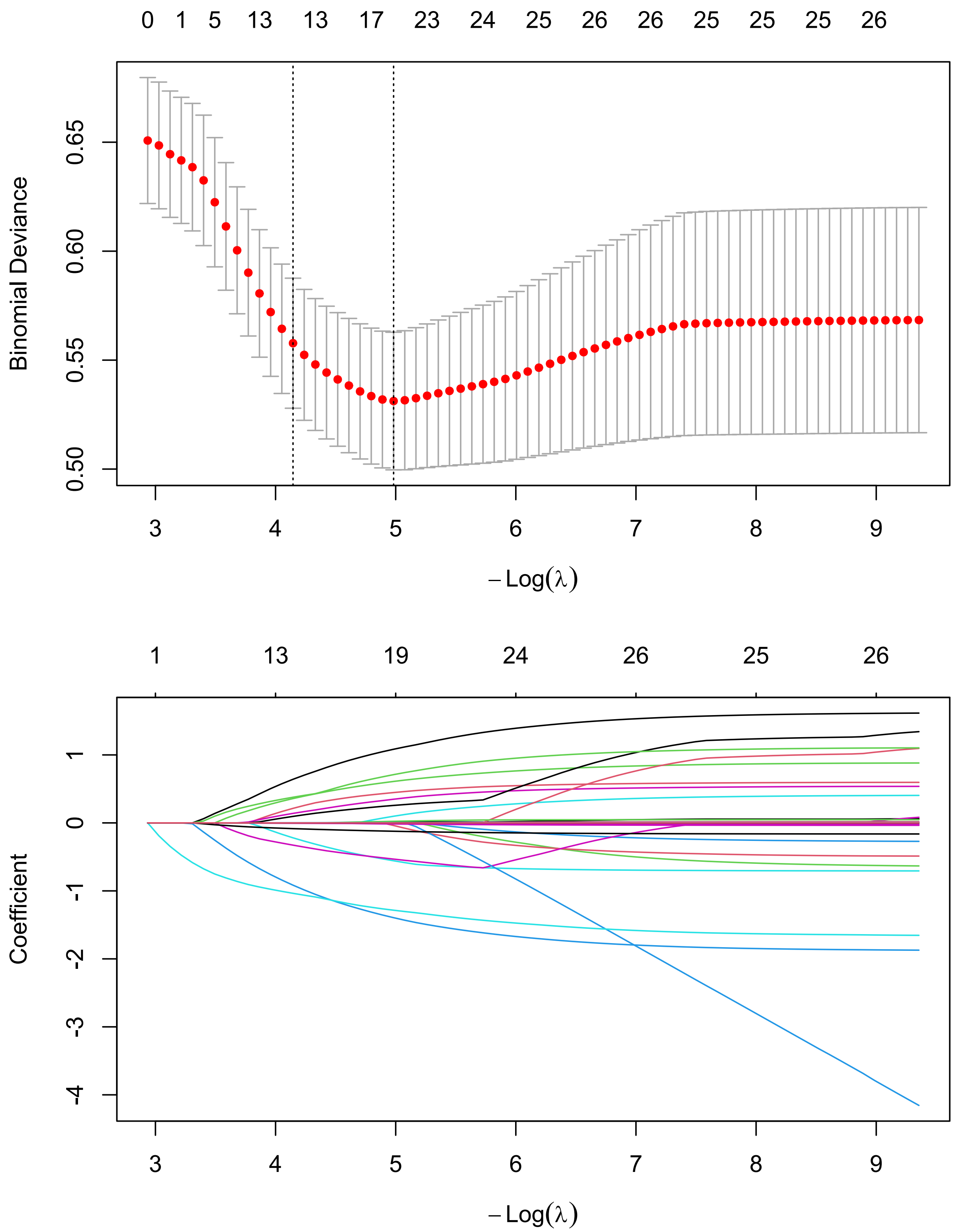

Figure S13. Feature selection using the least absolute shrinkage and selection operator (LASSO) regression. **(Top)** The binomial deviance is plotted against $-\log(\lambda)$. The red dots indicate the average deviance values for each model evaluated using 10-fold cross-validation, and the error bars represent standard errors. The vertical dotted lines define the optimal λ values (minimum deviance and 1 standard error of the minimum). **(Bottom)** The LASSO coefficient profiles of the clinical features are plotted against $-\log(\lambda)$. As the penalty factor increases (moving left on the x-axis), less important variables are continuously compressed to zero.

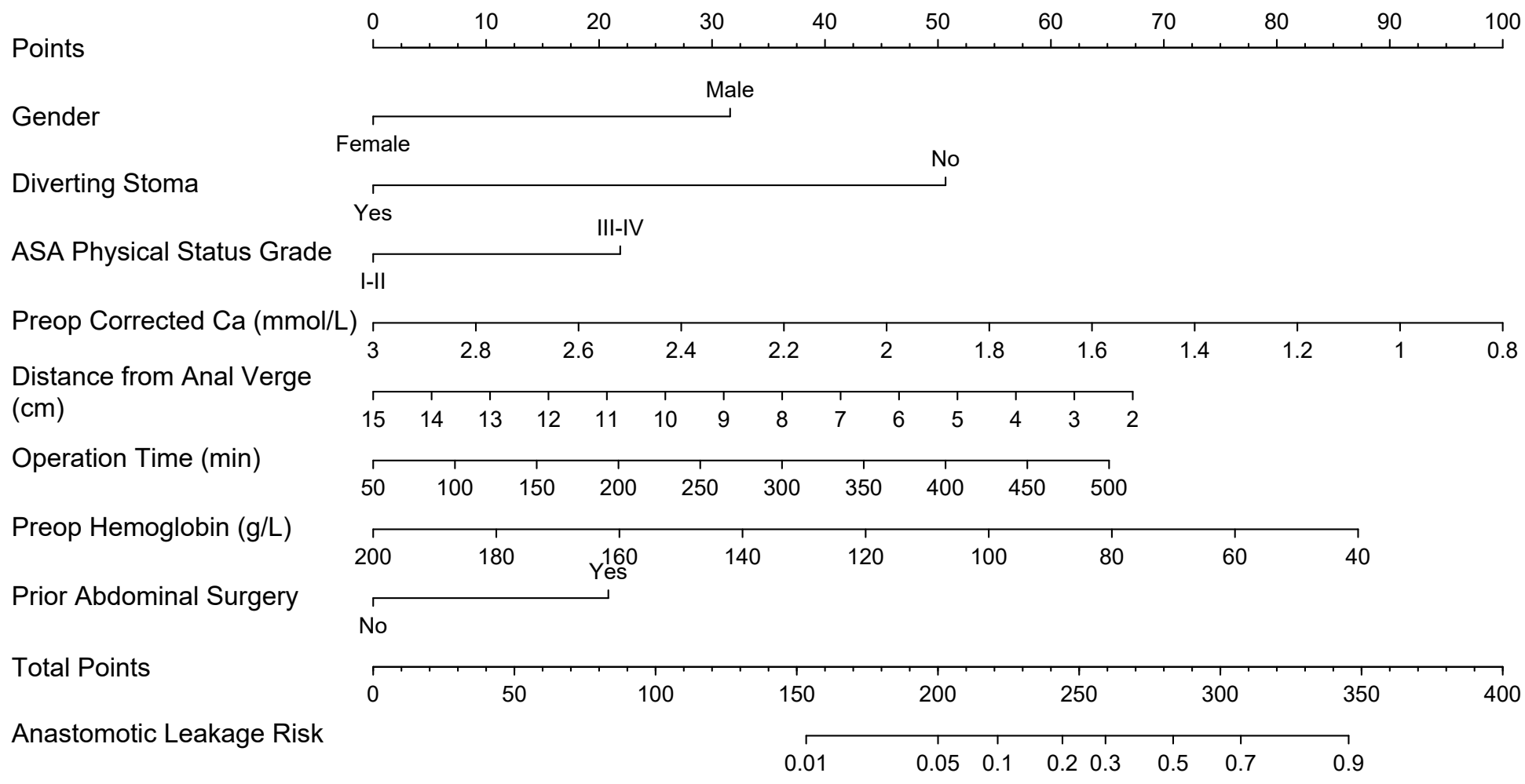

Model 1: Pre- and Intraoperative Baseline Nomogram

Figure S14. Nomogram for predicting the individualized risk of anastomotic leakage based on preoperative and intraoperative baseline factors (Model 1). To use the nomogram, locate the patient's specific value on each variable axis, and draw a vertical line upward to the "Points" axis to determine the score for that variable. Sum the scores from all variables to calculate the "Total Points". Finally, draw a vertical line downward from the "Total Points" axis to determine the estimated probability of anastomotic leakage.

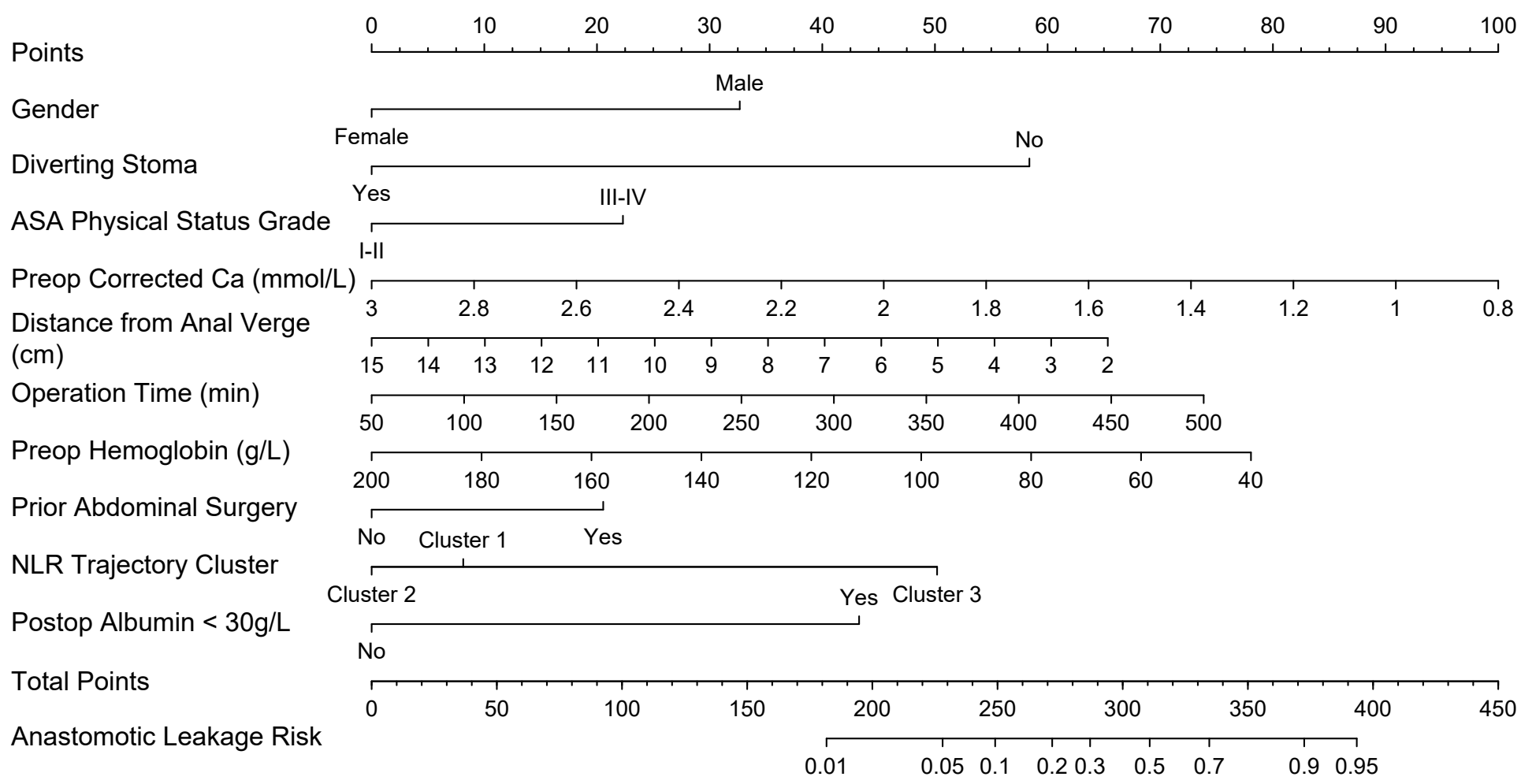

Model 2: Dynamic Sequential Nomogram Incorporating Postoperative Trajectories

Figure S15. Dynamic sequential nomogram incorporating early postoperative trajectories for the prediction of anastomotic leakage (Model 2). This enhanced model integrates baseline clinical features with early postoperative dynamic indicators (NLR trajectory cluster and postoperative albumin level on POD 3). The scoring methodology is identical to Model 1, allowing for a sequentially updated, individualized risk assessment tailored to the patient's postoperative clinical course.

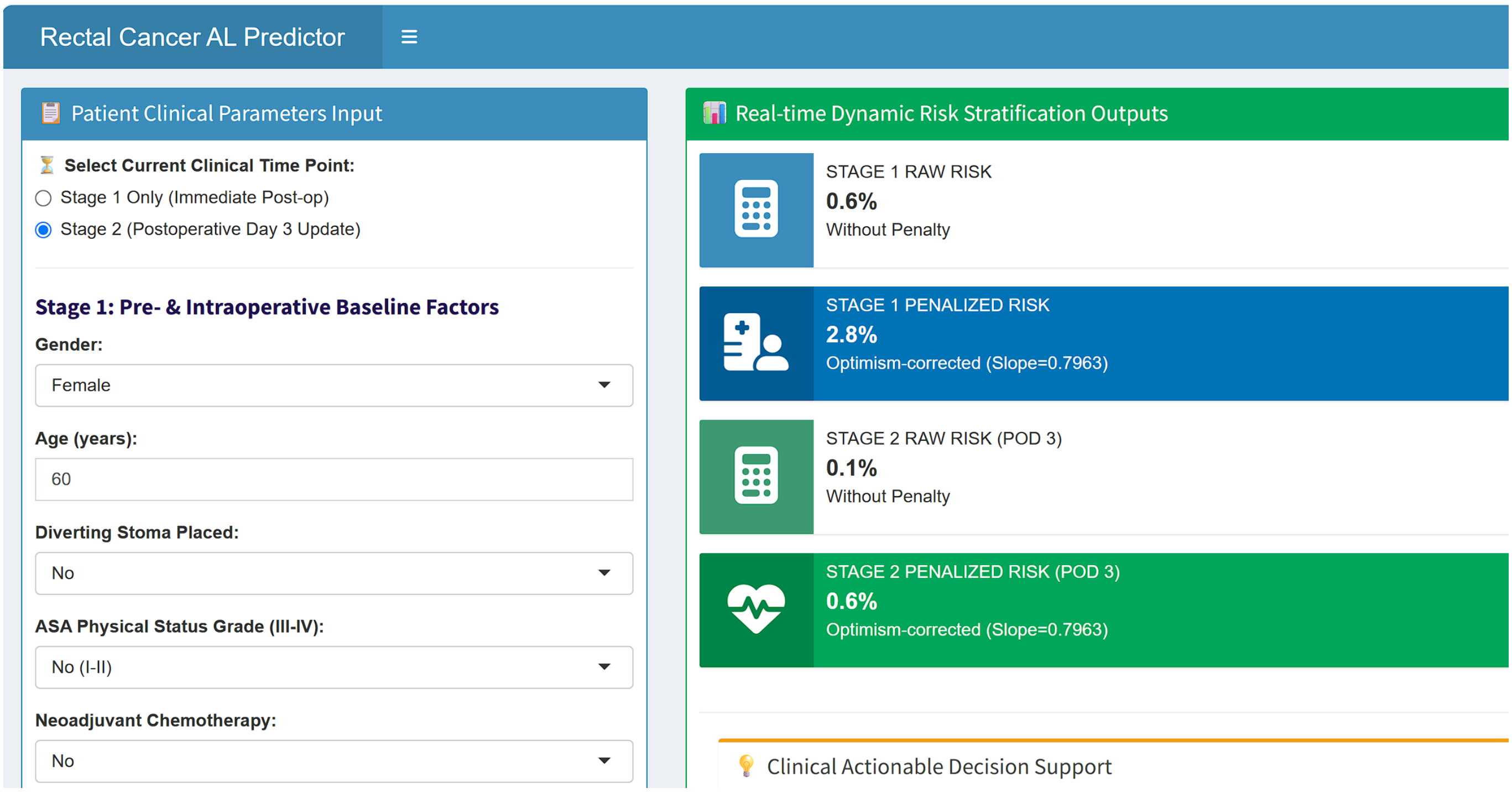

Figure S16. Interactive web-based dynamic risk calculator for real-time clinical decision support.The screenshot demonstrates the user interface of the definitive R Shiny application (`https://nsmc-al-prediction-model.shinyapps.io/AL_Predictor_Web/`). Unlike the parsimonious static nomograms, this comprehensive digital platform integrates the complete sets of 16 baseline and 19 sequential consensus predictors to maximize predictive accuracy. Clinicians can toggle between temporal decision points to input patient-specific parameters. Notably, to adhere to rigorous validation standards and mitigate potential overfitting, the system simultaneously outputs both raw probabilities and rigorously penalized risk estimates (adjusted via a uniform shrinkage factor based on the optimism-corrected calibration slope of 0.7963). Additionally, the tool provides actionable, algorithm-driven clinical recommendations tailored to the penalized dynamic risk profile, facilitating individualized perioperative management.

*TRIPOD 2024 Checklist for the Landmark-Based Dynamic Prediction Model for AL

Section/Topic	Item	D/E	Checklist item	Reported on page
**TITLE**				
Title	1	D; E	Identify the study as developing or evaluating the performance of a multivariable prediction model, the target population, and the outcome to be predicted	Page 1
**ABSTRACT**				
Abstract	2	D; E	See TRIPOD+AI for Abstracts checklist	Pages 1-2
**INTRODUCTION**				
Background	3a	D; E	Explain the healthcare context (including whether diagnostic or prognostic) and rationale for developing or evaluating the prediction model, including references to existing models	Pages 2-3
	3b	D; E	Describe the target population and the intended purpose of the prediction model in the context of the care pathway, including its intended users	Pages 2-3
	3c	D; E	Describe any known health inequalities between sociodemographic groups	Not applicable
Objectives	4	D; E	Specify the study objectives, including whether the study describes the development or validation of a prediction model (or both)	Page 3
**METHODS**				
Data	5a	D; E	Describe the sources of data separately for the development and evaluation datasets, the rationale for using these data, and the representativeness of the data	Page 3
	5b	D; E	Specify the dates of the collected participant data, including the start and end of participant accrual	Page 3
Participants	6a	D; E	Specify key elements of the study setting, including the number and location of centers	Page 3
	6b	D; E	Describe the eligibility criteria for study participants	Page 3

Continued on next page...

Continued from previous page

Section/Topic	Item	D/E	Checklist item	Reported on page
	6c	D; E	Give details of any treatments received and how they were handled during model development or evaluation, if relevant	Page 3
Data preparation	7	D; E	Describe any data pre-processing and quality checking	Pages 3-4
Outcome	8a	D; E	Clearly define the outcome that is being predicted and the time horizon	Page 3
	8b	D; E	If outcome assessment requires subjective interpretation, describe the qualifications of the outcome assessors	Page 3
	8c	D; E	Report any actions to blind assessment of the outcome to be predicted	Not applicable
Predictors	9a	D	Describe the choice of initial predictors and any pre-selection of predictors before model building	Page 4
	9b	D; E	Clearly define all predictors, including how and when they were measured	Pages 3-4
	9c	D; E	If predictor measurement requires subjective interpretation, describe the qualifications	Not applicable
Sample size	10	D; E	Explain how the study size was arrived at	Page 5
Missing data	11	D; E	Describe how missing data were handled. Provide reasons for omitting any data	Pages 3, 5
Analytical methods	12a	D	Describe how the data were used in the analysis, including whether the data were partitioned	Page 5
	12b	D	Depending on the type of model, describe how predictors were handled in the analyses	Pages 3-4
	12c	D	Specify the type of model, rationale, all model-building steps, including hyperparameter tuning, and method for internal validation	Pages 4, 5
	12d	D; E	Describe if and how any heterogeneity in estimates of model parameter values and model performance was handled	Not applicable
	12e	D; E	Specify all measures and plots used to evaluate model performance and compare multiple models	Page 5

Continued on next page...

Continued from previous page

Section/Topic	Item	D/E	Checklist item	Reported on page
	12f	E	Describe any model updating arising from the model evaluation	Not applicable
	12g	E	For model evaluation, describe how the model predictions were calculated	Page 4
Class imbalance	13	D; E	If class imbalance methods were used, state why and how this was done	Not applicable
Fairness	14	D; E	Describe any approaches that were used to address model fairness and their rationale	Not applicable
Model output	15	D	Specify the output of the prediction model	Pages 4, 6
Training vs eval	16	D; E	Identify any differences between the development and evaluation data	Page 5
Ethical approval	17	D; E	Name the institutional research board or ethics committee that approved the study	Page 10
**OPEN SCIENCE**				
Funding	18a	D; E	Give the source of funding and the role of the funders for the present study	Page 10
Conflicts of interest	18b	D; E	Declare any conflicts of interest and financial disclosures for all authors	Page 10
Protocol	18c	D; E	Indicate where the study protocol can be accessed or state that a protocol was not prepared	Not prepared
Registration	18d	D; E	Provide registration information for the study, or state that the study was not registered	Not registered
Data sharing	18e	D; E	Provide details of the availability of the study data	Page 10
Code sharing	18f	D; E	Provide details of the availability of the analytical code	Page 10
**PATIENT & PUB.**				
Involvement	19	D; E	Provide details of any patient and public involvement	No involvement
**RESULTS**				
Participants	20a	D; E	Describe the flow of participants through the study. A diagram may be helpful.	Page 4, Page 17 (Fig 1)

Continued on next page...

Continued from previous page

Section/Topic	Item	D/E	Checklist item	Reported on page
	20b	D; E	Report the characteristics overall and for each data source or setting. A table may be helpful.	Page 6, Pages 14-15 (Table 1)
	20c	E	For model evaluation, show a comparison with the development data of the distribution of important predictors.	Not applicable
Model development	21	D; E	Specify the number of participants and outcome events in each analysis.	Pages 5, 6
**Model specification**	**22**	**D**	**Provide details of the full prediction model to allow predictions in new individuals.**	**Pages 6, 7, Page 20 (Fig 4), Tables S5-S6**
Model performance	23a	D; E	Report model performance estimates with confidence intervals. Consider plots to aid the presentation.	Pages 7, 8, Page 18 (Fig 2)
	23b	D; E	If examined, report results of any heterogeneity in model performance across clusters.	Not applicable
Model updating	24	E	Report the results from any model updating.	Not applicable
**DISCUSSION**				
Interpretation	25	D; E	Give an overall interpretation of the main results, including issues of fairness in the context of the objectives and previous studies.	Pages 8, 9
Limitations	26	D; E	Discuss any limitations of the study and their effects.	Page 9
Usability	27a	D	Describe how poor quality or unavailable input data should be assessed and handled.	Not applicable
	27b	D	Specify whether users will be required to interact in the handling of the input data or use of the model.	Pages 8, 9
	27c	D; E	Discuss any next steps for future research, with a specific view to the applicability of the model.	Page 9

Notes: D = items relevant only to the development of a prediction model; E = items relating solely to the evaluation of a prediction model. ”Not applicable” indicates that the specific methodological step was not performed in this study.
