# Supplementary material for: A landmark-based dynamic prediction model for anastomotic leakage after rectal cancer surgery: integrating perioperative and postoperative trajectories
Source: Front Oncol. 2026 Sep 16;16:1915256. doi: 10.3389/fonc.2026.1915256 (PMC13623707; doi:10.3389/fonc.2026.1915256)
Supplement: Supplementary file 2 [file DataSheet2.zip › Fig.s5.pdf]

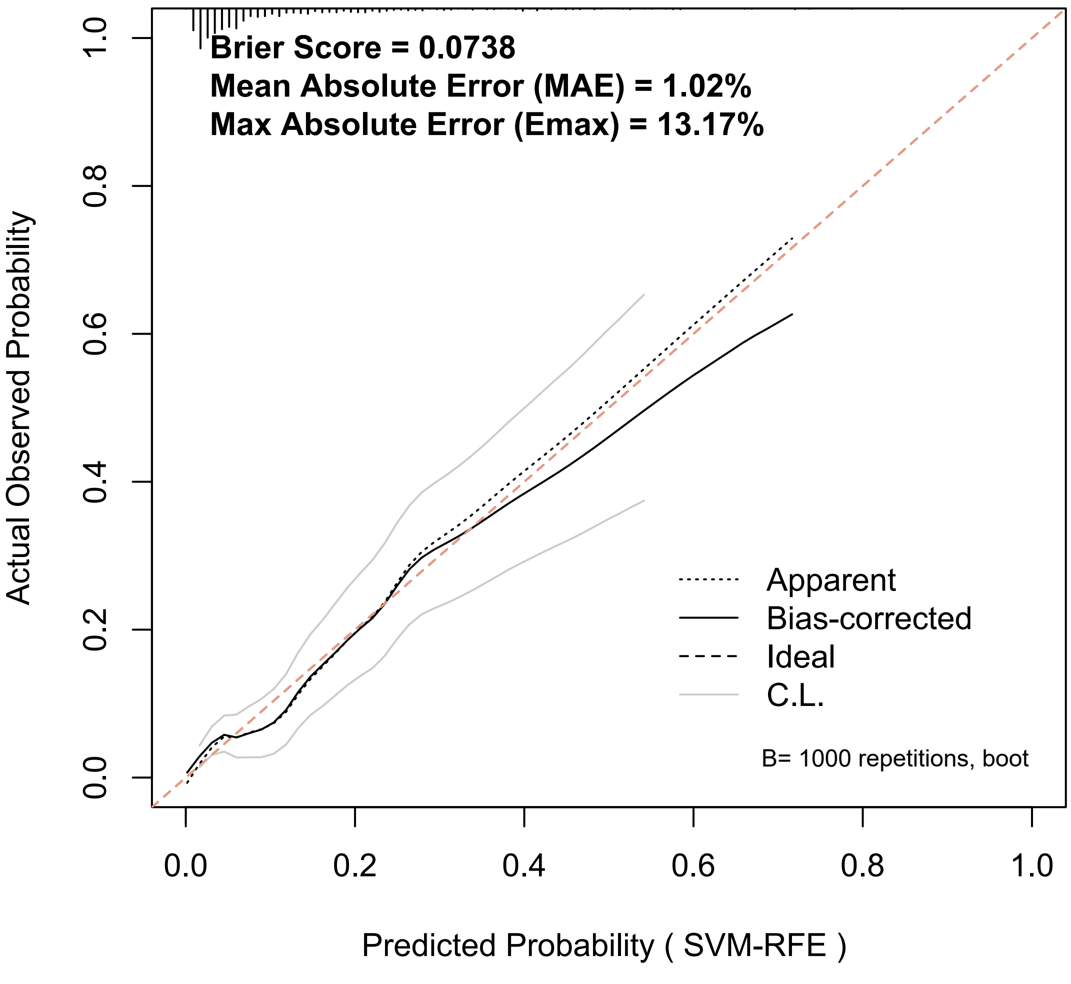

Brier Score = 0.0738
Mean Absolute Error (MAE) = 1.02%
Max Absolute Error (Emax) = 13.17%
Apparent
Bias-corrected
Ideal
C.L.
B= 1000 repetitions, boot
Actual Observed Probability
Predicted Probability (SVM-RFE)
0.0
0.2
0.4
0.6
0.8
1.0
