# Supplementary material for: A landmark-based dynamic prediction model for anastomotic leakage after rectal cancer surgery: integrating perioperative and postoperative trajectories
Source: Front Oncol. 2026 Sep 16;16:1915256. doi: 10.3389/fonc.2026.1915256 (PMC13623707; doi:10.3389/fonc.2026.1915256)
Supplement: Supplementary file 2 [file DataSheet2.zip › Fig.s16.pdf]

Patient Clinical Parameters Input

Stage 1: Pre- & Intraoperative Factors

Gender:

Female

Diverting Stoma Placed:

No

ASA Physical Status Grade:

I-II

Prior Abdominal Surgery History:

No

Preop Corrected Calcium (mmol/L):

2.25

Distance from Anal Verge (cm):

5

Operative Duration (minutes):

180

Preop Hemoglobin Concentration (g/L):

130

Stage 2: Postoperative Dynamic Indices (POD 3)

NLR Postoperative Trajectory Cluster:

Cluster 1

Postoperative Albumin < 30 g/L:

No

Real-time Dynamic Risk Stratification Outputs

STAGE 1 RISK (IMMEDIATE POST-OPERATION)

0.7%

STAGE 2 RISK (POSTOPERATIVE DAY 3 UPDATED)

0.4%

Clinical Actionable Decision Support

ROUTINE ACCELERATED RECOVERY PATHWAY:
The model predicts low cumulative dynamic probability of AL. Standard Fast-Track/ERAS protocols and gradual step-up diet can be safely maintained under standard ward surveillance.
