# Supplementary material for: A landmark-based dynamic prediction model for anastomotic leakage after rectal cancer surgery: integrating perioperative and postoperative trajectories
Source: Front Oncol. 2026 Sep 16;16:1915256. doi: 10.3389/fonc.2026.1915256 (PMC13623707; doi:10.3389/fonc.2026.1915256)
Supplement: Supplementary file 2 [file DataSheet2.zip › Fig.s15.pdf]

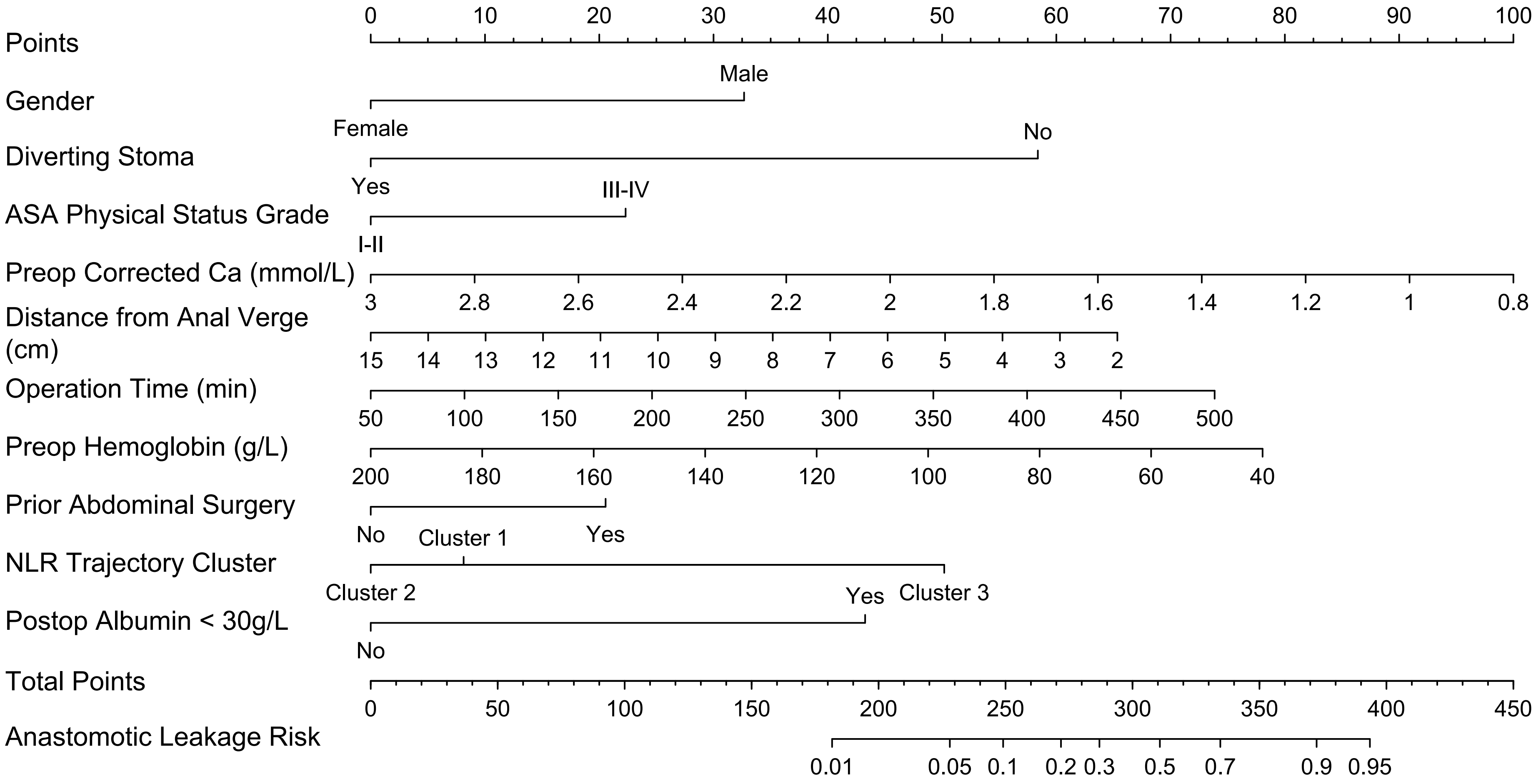

Model 2: Dynamic Sequential Nomogram Incorporating Postoperative Trajectories
