# Supplementary material for: A landmark-based dynamic prediction model for anastomotic leakage after rectal cancer surgery: integrating perioperative and postoperative trajectories
Source: Front Oncol. 2026 Sep 16;16:1915256. doi: 10.3389/fonc.2026.1915256 (PMC13623707; doi:10.3389/fonc.2026.1915256)
Supplement: Supplementary file 2 [file DataSheet2.zip › Fig.s13.pdf]

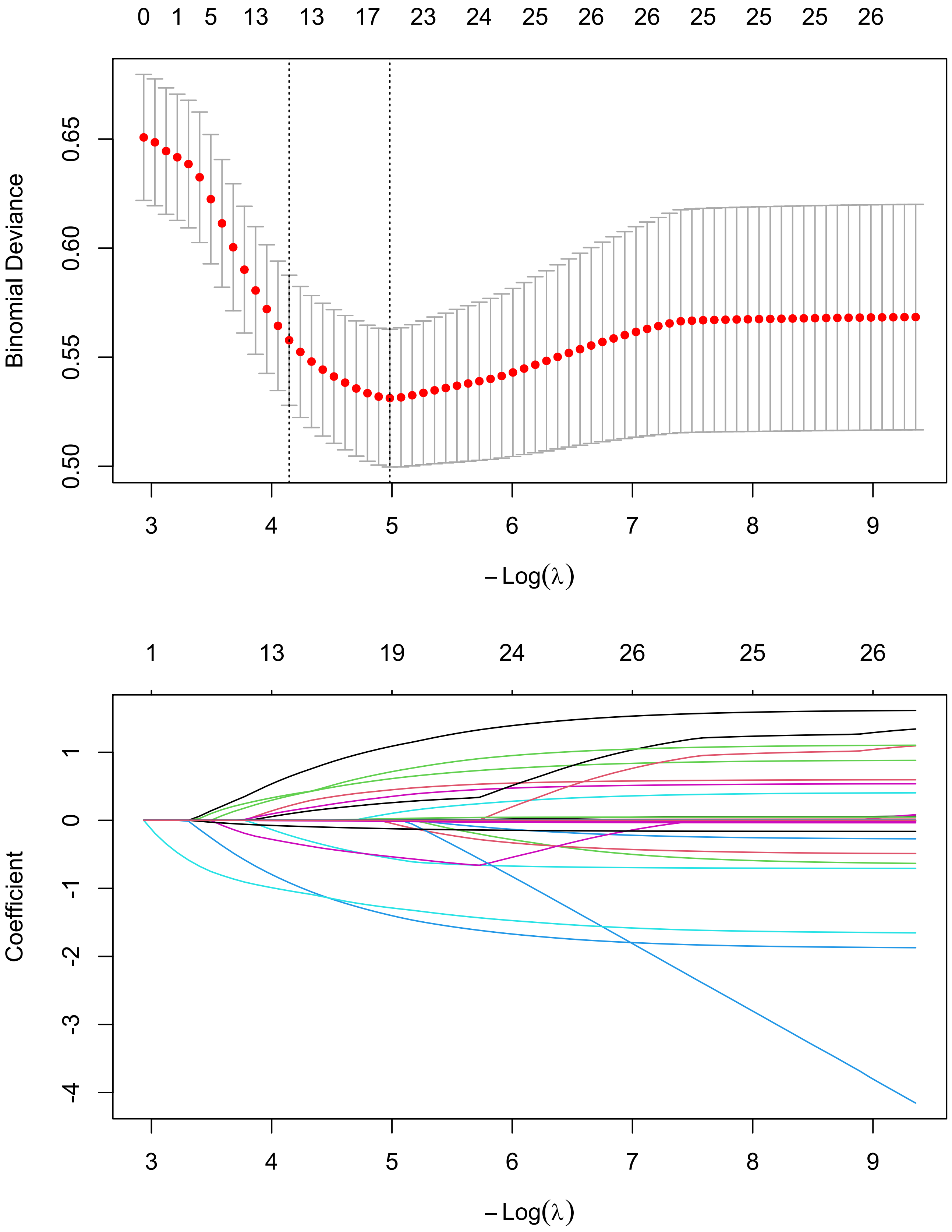
0 1 5 13 13 17 23 24 25 26 26 25 25 25 26
Binomial Deviance
0.50
0.55
0.60
0.65
3 4 5 6 7 8 9
– Log(λ)
1 13 19 24 26 25 26
Coefficient
-4
-3
-2
-1
0
1
3 4 5 6 7 8 9
– Log(λ)
