# Supplementary material for: A landmark-based dynamic prediction model for anastomotic leakage after rectal cancer surgery: integrating perioperative and postoperative trajectories
Source: Front Oncol. 2026 Sep 16;16:1915256. doi: 10.3389/fonc.2026.1915256 (PMC13623707; doi:10.3389/fonc.2026.1915256)
Supplement: Supplementary file 2 [file DataSheet2.zip › Fig.s12.pdf]

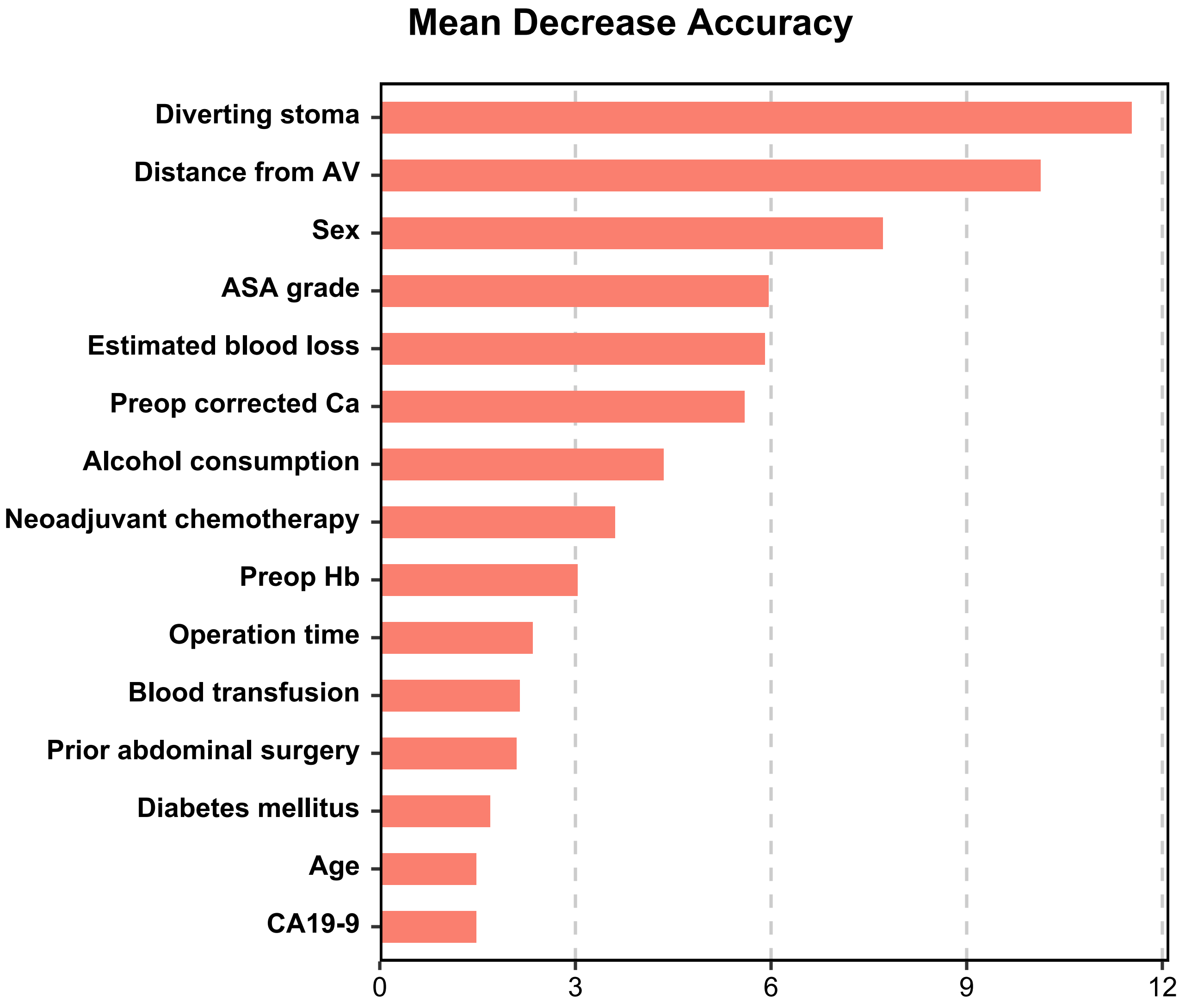
Mean Decrease Accuracy
Diverting stoma
Distance from AV
Sex
ASA grade
Estimated blood loss
Preop corrected Ca
Alcohol consumption
Neoadjuvant chemotherapy
Preop Hb
Operation time
Blood transfusion
Prior abdominal surgery
Diabetes mellitus
Age
CA19-9
0
3
6
9
12
