# Supplementary material for: A landmark-based dynamic prediction model for anastomotic leakage after rectal cancer surgery: integrating perioperative and postoperative trajectories
Source: Front Oncol. 2026 Sep 16;16:1915256. doi: 10.3389/fonc.2026.1915256 (PMC13623707; doi:10.3389/fonc.2026.1915256)
Supplement: Supplementary file 2 [file DataSheet2.zip › Fig.s11.pdf]

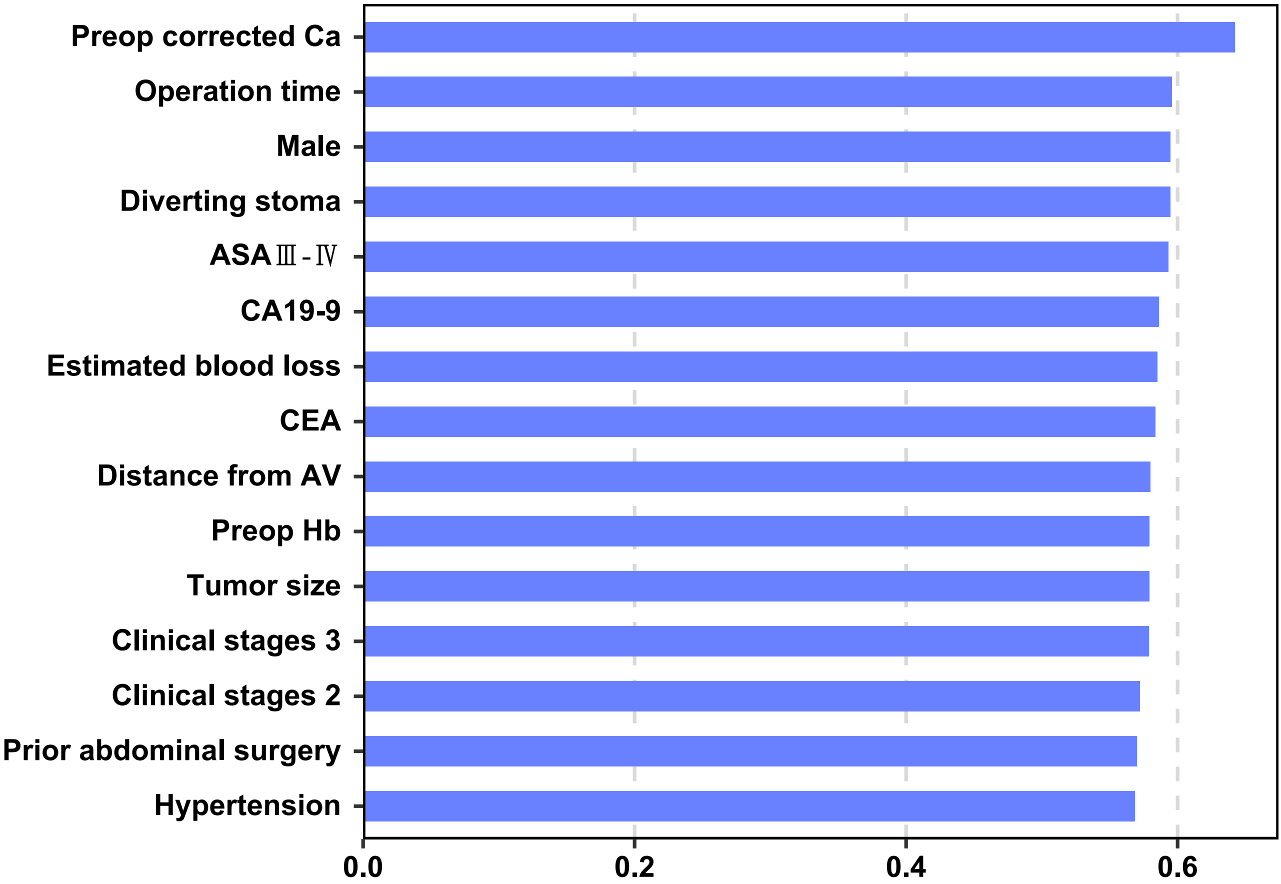
Preop corrected Ca
Operation time
Male
Diverting stoma
ASA Ⅲ - Ⅳ
CA19-9
Estimated blood loss
CEA
Distance from AV
Preop Hb
Tumor size
Clinical stages 3
Clinical stages 2
Prior abdominal surgery
Hypertension
0.0
0.2
0.4
0.6
