# Supplementary material for: A landmark-based dynamic prediction model for anastomotic leakage after rectal cancer surgery: integrating perioperative and postoperative trajectories
Source: Front Oncol. 2026 Sep 16;16:1915256. doi: 10.3389/fonc.2026.1915256 (PMC13623707; doi:10.3389/fonc.2026.1915256)
Supplement: Supplementary file 2 [file DataSheet2.zip › Fig.s10.pdf]

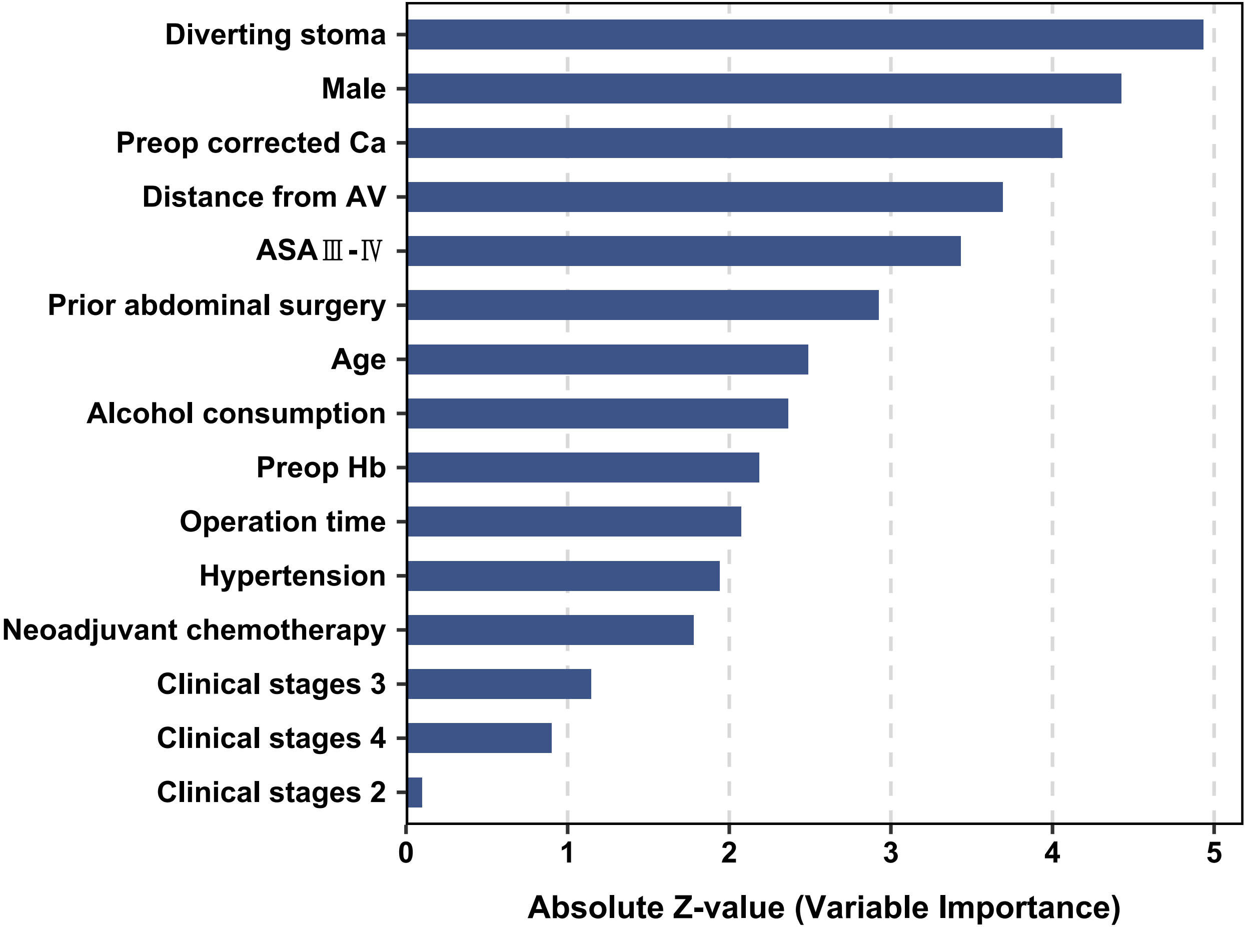
Diverting stoma
Male
Preop corrected Ca
Distance from AV
ASA Ⅲ - Ⅳ
Prior abdominal surgery
Age
Alcohol consumption
Preop Hb
Operation time
Hypertension
Neoadjuvant chemotherapy
Clinical stages 3
Clinical stages 4
Clinical stages 2
0
1
2
3
4
5
Absolute Z-value (Variable Importance)
