# Supplementary material for: A landmark-based dynamic prediction model for anastomotic leakage after rectal cancer surgery: integrating perioperative and postoperative trajectories
Source: Front Oncol. 2026 Sep 16;16:1915256. doi: 10.3389/fonc.2026.1915256 (PMC13623707; doi:10.3389/fonc.2026.1915256)
Supplement: Supplementary file 2 [file DataSheet2.zip › Fig.s1.pdf]

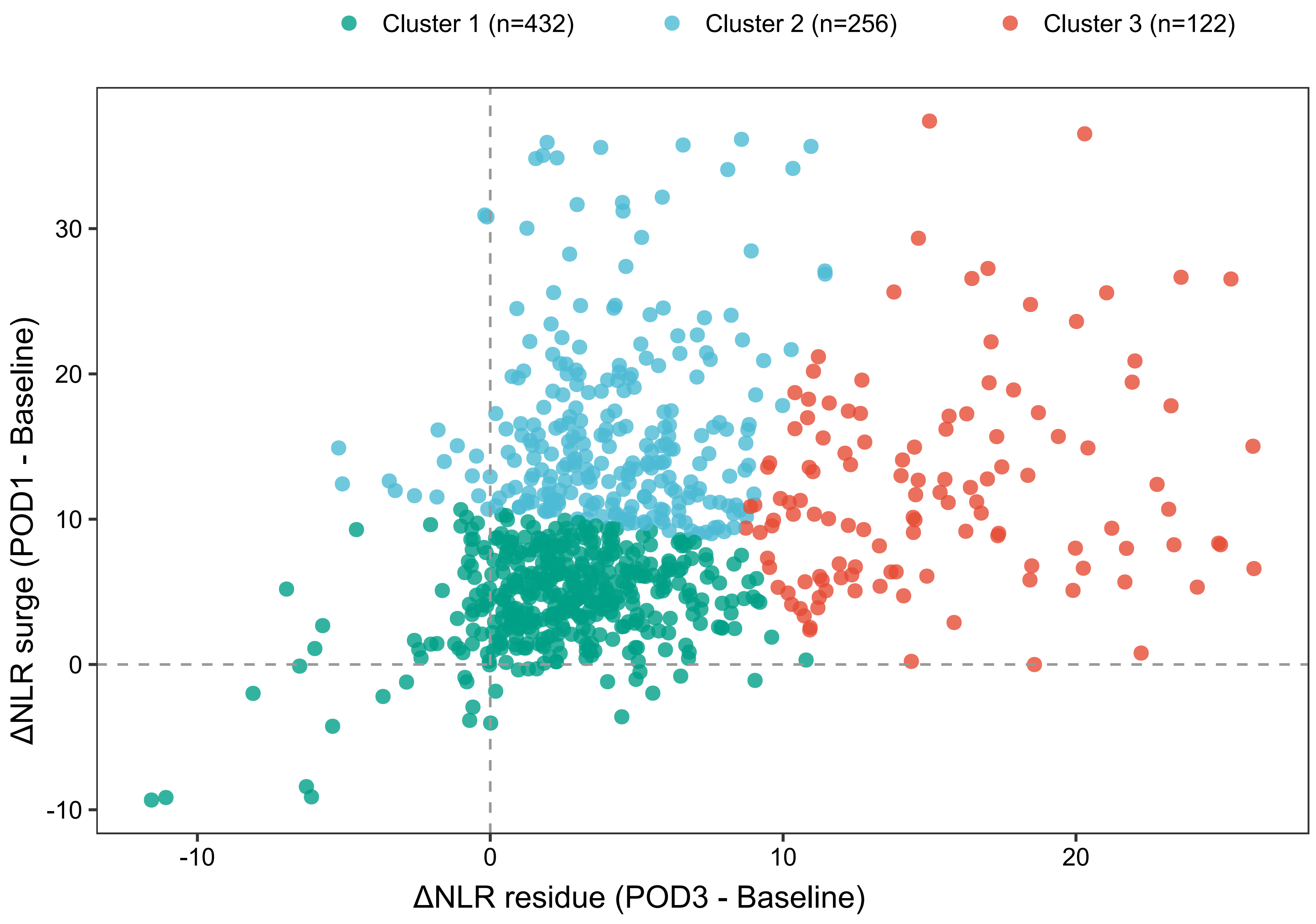
Cluster 1 (n=432)
Cluster 2 (n=256)
Cluster 3 (n=122)
ΔNLR surge (POD1 - Baseline)
30
20
10
0
-10
-10
0
10
20
ΔNLR residue (POD3 - Baseline)
